# Optical Spin Angular Momentum: Properties, Topologies, Detection and Applications

**DOI:** 10.3390/nano15231798

**Published:** 2025-11-28

**Authors:** Shucen Liu, Xi Xie, Peng Shi, Yijie Shen

**Affiliations:** 1 Nanophotonics Research Center, Institute of Microscale Optoelectronic & State Key Laboratory of Radio Frequency Heterogeneous Integration, Shenzhen University, Shenzhen 518060, China; 2 Centre for Disruptive Photonic Technologies, School of Physical and Mathematical Sciences, Nanyang Technological University, Singapore 637371, Singapore; 3 School of Electrical and Electronic Engineering, Nanyang Technological University, Singapore 639798, Singapore

**Keywords:** angular momentum, spin-momentum locking, topological structure, optical differential computing, optical detection, transverse optical force

## Abstract

Spin angular momentum is a fundamental dynamical property of elementary particles and fields, playing a critical role in light–matter interactions. In optical studies, the optical spin angular momentum is closely linked to circular polarization. Research on the interaction between optical spin and matter or structures has led to numerous novel optical phenomena and applications, giving rise to the emerging field of spin optics. Historically, researchers primarily focused on longitudinal optical spin aligned parallel to the mean wavevector. In recent years, investigations into the spin–orbit coupling properties of confined fields—such as focused beams, guided waves, and evanescent waves—have revealed a new class of optical spin oriented perpendicular to the mean wavevector, referred to as optical transverse spin. In the optical near-field, such transverse spins arise from spatial variations in the momentum density of confined electromagnetic waves, where strong coupling between spin and orbital angular momenta leads to various topological spin structures and properties. Several reviews on optical transverse spin have been published in recent years, systematically introducing its fundamental concepts and the configurations that generate it. In this review, we detail recent advances in spin optics from three perspectives: theory, experimental techniques, and applications, with a particular emphasis on the fundamental physics of transverse spin and the resulting topological structures and characteristics. The conceptual and theoretical framework of spin optics is expected to significantly support further exploration of optical spin-based applications in fields such as optics imaging, topological photonics, metrology, and quantum technologies. Furthermore, these principles can be extended to general classical wave systems, including fluidic, acoustic, and gravitational waves.

## 1. Introduction

Spin angular momentum (SAM), as a fundamental dynamical physical quantity of elementary particles and classical wave fields, holds profound fundamental research significance in fields such as condensed matter physics, particle physics, and nuclear physics. It has led to numerous applications including nuclear magnetic resonance, giant magnetoresistance effect, electron spin resonance spectroscopy, and spin-transfer torque [1,2,3,4,5,6]. In research within these domains, investigators not only focus on the fundamental properties of SAM but also seek more flexible methods for its manipulation under various conditions. A commonly employed manipulation strategy involves the application of external magnetic fields, achieved by controlling the field’s strength or orientation. However, these methods exhibit several limitations, such as material’s properties, non-local nature, and high energy consumption, which consequently restrict their scope of applications [7,8].

Furthermore, light exhibits particle properties and thus possesses angular momentum (AM). The SAM of light is intimately related to the circular polarization state of the optical field [9]. In recent years, a growing number of researchers have gradually shifted their attention toward the manipulation of optical SAM [10,11,12,13,14]. With the rapid advancement of modern micro–nano fabrication technologies, researchers can more easily and flexibly control multiple degrees of freedom of light. However, at the nanoscale, classical degrees of freedom of light such as intensity, phase, and polarization are coupled and interact with each other, making their independent and flexible manipulation relatively challenging. In this context, the control of SAM offers certain advantages over the manipulation of other degrees of freedom. For example, through spin–orbit interactions [15,16,17], SAM and orbital AM (OAM) can be manipulated independently, enabling more precise manipulation and utilization of the spin properties of photons without interference from other optical parameters. The numerous advantages of SAM open up new possibilities for photonics, particularly in optical manipulation [18,19,20,21,22,23,24,25,26,27,28], sensing [29,30,31,32,33,34,35], information processing [36,37,38,39,40,41,42,43,44,45,46,47,48], chiral quantum optics [49,50,51,52,53,54,55,56,57], and quantum entanglement [58,59,60,61,62,63,64,65,66,67].

In recent years, significant progress has been made in the manipulation techniques associated with SAM. These advances primarily involve the engineering of optical field configurations, structural designs, and material properties. Concurrently, the control over SAM has also accelerated research in optical encryption, chiral sensing, and optical communications [68,69,70,71,72,73,74,75,76,77]. It is noteworthy that a considerable portion of past work focused only on the longitudinal component of SAM, i.e., the component parallel to the wavevector **k** [9]. In reality, SAM possesses a structured nature and includes a transverse spin component perpendicular to the wavevector [78,79]. For a long time, these transverse components received limited attention, and their unique properties—such as the helix-dependent nature of transverse spin, its origin, and its spin-momentum locking behavior—remained unexplored [10,11,12,13,14]. To achieve full-dimensional control of SAM, theoretical extensions in this area have naturally become a focus of research. In 2015, Konstantin Y. Bliokh and colleagues at the RIKEN Institute in Japan leveraged the formal similarity between the Dirac and Maxwell equations to propose, in a theoretical paper published in Science, the “Quantum spin Hall effect of light”. This work introduced for the first time the concept of an intrinsic transverse spin-momentum locking (SML) in optical fields [78], attracting widespread interest from researchers worldwide. However, it is important to note that the transverse spin has a spin Chern number C_spin_ = 4, meaning this “Quantum spin Hall effect” lacks topological protection. Therefore, we refer to this property as the intrinsic spin-momentum locking of light (iSML) [80]. The above iSML was initially proposed for surface plasmon polariton waves. In 2021, Shi et al. at Shenzhen University proposed a generalized iSML relation for photonic transverse spin [15], which describes the relationship between transverse spin and Poynting momentum in complex structured evanescent waves and reveals the origin of transverse spin. Subsequently, this generalized iSML framework has been extended to general optical fields [17], dispersive media [16], and randomly polarized light [81], demonstrating potential applications in chiral manipulation, integrated optics, and optical communications.

Following these developments, researchers focused on the generation and manipulation of novel structured optical fields based on SAM, leading to progress in classical optics, topological photonics, and quantum optics. Discoveries include spin structures such as photonic skyrmions [82,83,84,85,86,87] and topological merons [88,89,90,91]. The discovery of these spin structures relies on the development of SAM detection techniques. A review of recent work shows that SAM detection is no longer confined solely to near-field or far-field measurements alone. The most conventional detection method relies on assessing the optical chirality [92], but this approach can only detect the longitudinal spin component of the field. For complex optical fields—such as those supported by surface plasmon polaritons, waveguides, or scattering structures that carry transverse spin—new detection strategies are required.

For example, detecting the SAM in surface plasmon polaritons typically involves near-field scanning optical microscopy (NSOM). Probe-based NSOM systems can effectively extract desired electromagnetic field components, with resolution flexibly controlled through probe design [93]. Alternatively, scattering particles made from tailored materials and sizes can also perform such detection with additional functional capabilities [94,95,96,97,98]. In recent years, several far-field detection techniques have attracted attention, such as methods employing nonlinear effects to convert near-field plasmonic information into far-field signals that can be captured using imaging elements [99].

**Figure 1:**
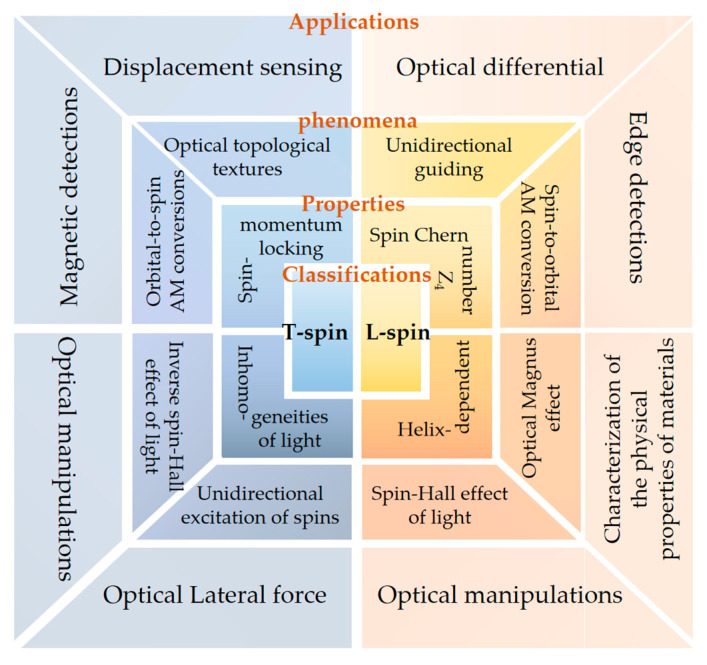
The categories, related properties, phenomena, and application fields of optical AM.

The detection of SAM must account not only for individual components but also multi-dimensional coupling and full-component reconstruction [100,101,102,103,104]. Significant advances have been made in these directions, providing new pathways for controlling SAM and thereby promoting developments in high-precision optical sensing, computational imaging, optical communications, and quantum communications (Figure 1).

This article reviews recent research advances in the manipulation of SAM from the perspectives of its underlying mechanisms, detection techniques, and practical applications. It also offers prospects for future developments in terms of full-dimensional control, super-resolution, and flexibility within the field. Additionally, we emphasize the significant role of research on SAM in promoting interdisciplinary convergence involving optics, biology, materials science, and medicine. In summary, by synthesizing progress in this area, we aim to provide a comprehensive reference to support future scientific research and industrial development.

## 2. Mechanisms of Optical Spin Angular Momentum Manipulation

### 2.1. Momentum Density and Angular Momentum Density of Light

Light exhibits wave–particle duality, with its particle nature being manifested through the momentum and AM of the optical field. We consider monochromatic light fields where the instantaneous field vectors depend on time *t* harmonically. Following the usual representation, electric and magnetic vectors are described as 𝓔(*t*) = Re[**E**$e^{-i\omega t}$] and 𝓗(*t*) = Re[**H**$e^{-i\omega t}$], where *ω* is the angular frequency, 𝓔 and 𝓗 are complex valued quantities, time-independent or varying very slowly with respect to the oscillation period. Then, the Poynting vector, averaged over the oscillation period, can be presented as P=Re(E*×H)/2. The study of momentum can be described using the Poynting vector [105,106,107]; however, considering observability and relevance, researchers tend to prefer the orbital (or canonical) momentum density po=Po/v2=Im(ε[E*·(∇)E]+μ[H*·(∇)H])/4ω. The OAM density can then be derived from the cross product of the position vector and the orbital momentum density ($L=r\times p^{o}$). OAM can be categorized as either intrinsic or extrinsic. Intrinsic OAM (i-OAM) is intimately linked to the vortex phase, whereas extrinsic OAM (e-OAM) is associated with the trajectory of the light beam [108,109,110,111,112]. In contrast, SAM typically embodies the circular polarization characteristics of an optical field [113,114,115,116] and is given directly by the relation S=Im(εE*×E+μH*×H)/4ω.

Indeed, the aforementioned description provided by classical optical theory is consistent with the description obtained using the photon wave function [117,118,119,120,121,122]. To illustrate the relationship between momentum density and AM density, for instance, one can introduce the wave function to calculate the momentum density in the case of a uniform medium. Here, the wave function is expressed as

(1) $$|\psi \rangle =\frac{1}{2}\left(\begin{array}{c} \sqrt{\varepsilon }E\\ i\sqrt{\mu }H \end{array}\right)$$

Then, the Poynting momentum density, canonical momentum density, spin-momentum density, SAM density, and OAM density can be, respectively, expressed as

(2) $$p=\frac{1}{\hbar \omega }\langle \psi |\hat{p}(r)|\psi \rangle +\nabla \times \frac{1}{2\hbar \omega }\langle \psi |\hat{S}|\psi \rangle =p^{o}+p^{s}$$

(3) po=14ωImε[E*·(∇)E]+μH*·(∇)H, ps=12∇×S

(4) S=1ℏωψS^ψ=14ωImεE*×E+μH*×H, L=r×po

Here, the momentum operator $\hat{p}(r)=-i\hbar \nabla$, acts on the spatial distribution of the wave function, while the SAM operator $\hat{S}$ is given by the spin-1 matrices [116], which act on the vectorial degrees of freedom in the complex three-dimensional space. Consequently, the two terms in Expression (2) correspond to the scalar field and the vector field, respectively. Furthermore, it is noted that the momentum density in Equation (2) consists of two parts: $p^{o}$ and $p^{s}$. The former represents the orbital momentum density, which arises from the inhomogeneity of the optical field’s phase [12]. The latter denotes the spin-momentum density, a quantity introduced by F. J. Belinfante to explain quantum spin and to symmetrize the canonical energy-momentum tensor in field theory [123]. It is analogous to the boundary magnetization current in solid-state-like systems or the topological quantum Hall current.

**Figure 2:**
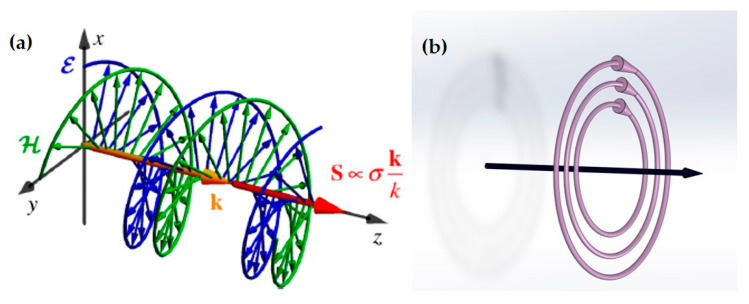
Momentum and spin in circularly polarized plane waves [114]: (**a**) Instantaneous electric and magnetic fields during the propagation of circularly polarized (m = i) plane wave. (**b**) The black and purple vector arrows represent the orbital momentum density and spin-momentum density, respectively.

It is worth noting that the introduction of spin-momentum density $p^{s}$ is of significant importance. For instance, when discussing the momentum and AM density of a circularly polarized plane wave (in the absence of boundaries), the SAM density S≠0, and S are proportional to the polarization ellipticity σ, which can be further derived $p^{s}\neq 0$ from Equation (3). However, in practical terms, a circularly polarized plane wave is a scalar field, leading to a zero spin-momentum density $p^{s}=0$. This appears inconsistent with the previous description. To resolve this apparent inconsistency, a hypothesis $p^{s}$ can be proposed: the plane wave is assumed to be composed of an infinite array of vortex currents. The cancelation between adjacent vortex currents results in a net zero spin-momentum density (σ=0), while simultaneously yielding a non-zero SAM density. This seemingly paradoxical situation, where a circularly polarized plane wave possesses non-zero SAM density yet zero spin momentum, lies at the heart of the well-known “plane-wave angular momentum paradox”. This paradox, concerning the decomposition of light’s angular momentum and its manifestation in confined beams, has been extensively analyzed in the literature [124,125,126] (see Figure 2).

Similarly, the total AM density comprises both orbital and spin components and can be expressed as J=L+S, generated by the terms corresponding to the momentum density, respectively. The OAM density can be obtained from $L=r\times p^{o}$, indicating that it is an extrinsic property of light (dependent on the choice of origin). The SAM density is given by S=Im(εE*×E+μH*×H)/4ω, showing that it is an intrinsic property (independent of the choice of origin). However, it is important to note that the net SAM, obtained by spatially integrating the spin-momentum density, satisfies $\langle S\rangle =\int S\mathrm{dV}=\int r\times p^{s}\mathrm{dV}$ for a plane wave.

### 2.2. Spin Angular Momentum of Light

#### 2.2.1. Longitudinal Spin in Paraxial Optical Fields

Introducing the theory of AM can begin with paraxial beams in free space. Consider an elliptically polarized vortex beam propagating along the *z*-axis direction, ignoring the small longitudinal electromagnetic field components (*z*-components). Its complex amplitude form can be expressed as

(5) $$E=A(\rho ,z)\frac{\bar{x}+m\bar{y}}{\sqrt{1+{\left|m\right|}^{2}}}\exp(ikz+il\varphi ),H=\bar{z}\times E$$

Here, A(ρ,z) represents the spatial distribution of the amplitude, the complex parameter m characterizes the polarization state of the beam [12]. When the beam is elliptically polarized, the polarization ellipticity is σ=2Im(m/(l+m2)), *l* is the topological charge of the vortex beam, and the propagation wavevector is k=ω/c, *c* being the light velocity.

Substituting Equation (5) into Equation (4), the SAM density can be further obtained:

(6) $$S=\frac{W}{\omega }\sigma \bar{z}$$

Here, $W=(\varepsilon |A{|}^{2})/2$ is the energy density. It can be seen that the SAM density S depends only on the polarization ellipticity σ and is a quantity independent of the external origin position, making it an intrinsic property of light.

This discussion concerns the density of AM. For a beam with finite boundaries, the spatial integral of the density can be used to quantify its characteristics. The integral of the AM density over the cross-section perpendicular to the propagation direction *x*-*y* is given below:

(7) $$\langle S\rangle \propto \sigma \frac{\langle k\rangle }{k}$$

Here, $\langle k\rangle$ denotes the energy density weighted average wavevector of the field’s angular spectrum, representing the local wavevector and the propagation direction of the wave packet. For the SAM, its magnitude is still closely related to the polarization ellipticity σ, and its direction is parallel to the direction of the wavevector **k**. Therefore, for a paraxial circularly polarized beam in free space, it is generally considered to carry longitudinal spin (parallel to **k**). The mutual coupling and interaction between longitudinal spin and matter, as well as orbital angular momentum, give rise to a series of novel phenomena, including the spin Hall effect, optical Magnus effect, spin–orbit angular momentum conversion, and so on [127,128,129,130,131,132,133,134,135,136,137,138,139,140,141,142,143,144,145,146,147]. It is noteworthy that in strongly focused light beams, the SAM of circularly polarized light can convert into OAM, manifesting as a vortex structure in the longitudinal component of the electric field, with its topological charge determined by the initial polarization and phase singularity [148,149,150]. However, during the research on SAM, researchers discovered another type of spin component orthogonal to the wavevector **k**, known as transverse spin. This spin typically originates from the rotation of the polarization state within the *x*-*z* plane. From the perspective of electromagnetic field quantities, it can be interpreted as the existence of electromagnetic field components parallel to **k** that have a phase difference with components orthogonal to **k**. The following sections will introduce several typical optical fields possessing transverse spin, including evanescent fields and interference fields. Since focused fields can be considered a special case of interference fields, they will not be discussed in depth later.

#### 2.2.2. Transverse Spin in Evanescent Fields

**Figure 3:**
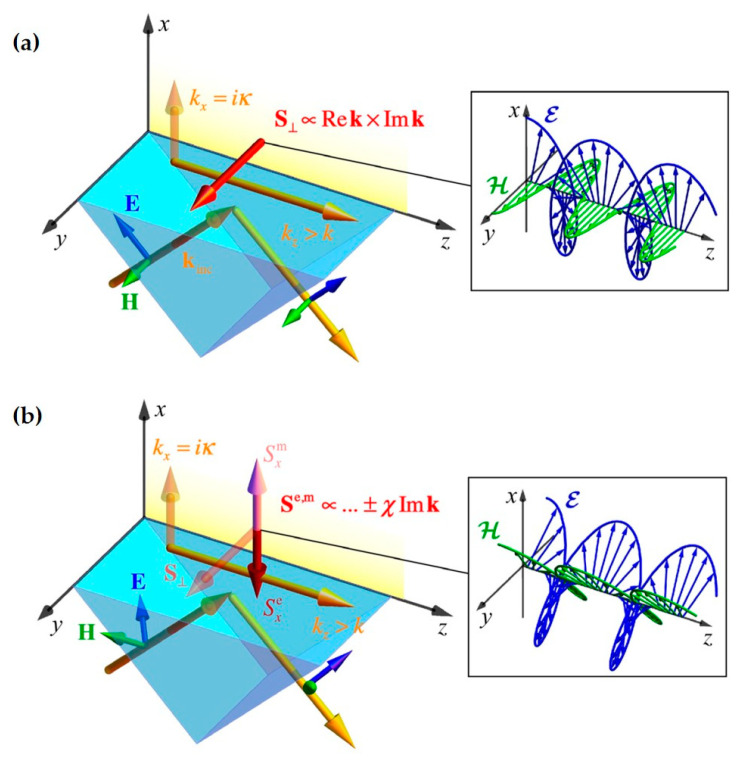
The evanescent wave generated by total internal reflection has a transverse spin angular momentum (t-SAM) density [12]: (**a**) the inset shows the *z*-evolution of the instantaneous electric and magnetic fields **ℰ** and **ℋ** for the simplest linear x-polarization; (**b**) when the polarization direction is along ±45, the generated evanescent wave has a t-SAM density $S^{e,m}$, which is the result of the combined effect of electric and magnetic fields.

Before discussing evanescent fields, assume a wave propagating along the interface with arbitrary polarization, as shown in Figure 3a, decaying in the half-space *x* > 0. Its complex amplitude form can be expressed as

(8) $$E=\frac{A_{0}}{\sqrt{1+{\left|m\right|}^{2}}}(\bar{x}+m\frac{\kappa }{k_{z}}\bar{y}-i\frac{\kappa }{k_{z}}\bar{z})\exp(ik_{z}-\kappa x),H=\frac{k}{k}\times E$$

Here, m is used to describe the polarization state of the beam. It is linear polarization when m=0/∞, and circular polarization when m=i/−i. The case m=0 is considered here. $k_{z}$ and iκ are the wavevectors in the *z* and *x* directions, respectively, where κ describes the decay of the evanescent wave, and *k* is the propagation wavevector in free space. They satisfy $k=k_{z}+i\kappa$ and $k^{2}={k_{z}}^{2}+\kappa^{2}$. From Equation (8), it can be seen that the $E_{z}$ and $E_{x}$ components of the electric field differ by an imaginary unit i, which means their relative phase shift of ±π/2 and causes the electric field polarization to rotate in the *x*-*z* plane, generating the transverse component $S_{y}$ of the SAM density. From Equations (4) and (8), we obtain Sy∝Im(Ez*Ex+Hz*Hx). The longitudinal electric field component $E_{z}$ is crucial for the generation of the t-SAM density. From the imaginary component $-i\frac{\kappa }{k_{z}}\bar{z}$, it is clear that only when there is a decay term wavevector iκ does a phase lag between $E_{z}$ and $E_{x}$ exist, revealing why evanescent fields can generate t-SAM density. It is emphasized that the above analysis considers the case of transverse magnetic (TM) mode generating t-SAM density [151]. In fact, the transverse electric (TE) mode (m=∞,TE) can also generate t-SAM density, but the difference is that the former is caused by the rotation of the electric component in the *x*-*z* plane, while the latter is caused by the rotation of the magnetic component in the *x*-*z* plane [91].

Whether TE or TM mode, they consider the case of purely linearly polarized evanescent waves. In this case, the optical field does not possess a longitudinal spin angular momentum (l-SAM) density. However, actual optical fields are often complex, requiring consideration of arbitrarily polarized evanescent waves. Below, the SAM density for an arbitrarily polarized evanescent wave is given. First, the normalized Stokes parameter description of an arbitrary polarization state is provided:

(9) τ=1−m21+m2χ=2Rem1+m2σ=2Imm1+m2

These three parameters describe the 0°/90° linear polarization state (τ=±1), the 45°/−45° linear polarization state (χ=±1), and the left-handed/right-handed circular polarization state (σ=±1), respectively, and they satisfy $\tau^{2}+\chi^{2}+\sigma^{2}=1$.

(10) $$S^{e,m}\propto \frac{W}{2\omega }(\sigma \frac{k}{k_{z}}\bar{z}+(1\pm \tau )\frac{\kappa }{k_{z}}\bar{y}\pm \chi \frac{\kappa k}{{k_{z}}^{2}}\bar{x})$$

It can be seen that the SAM density of the evanescent field generated by an arbitrarily polarized beam consists of three parts: the l-SAM density $S_{z}$ related to the polarization ellipticity σ; the t-SAM density $S_{y}$ related to κ and τ; and the t-SAM density $S_{x}$ related to κ and χ, where $S_{x}$ is also orthogonal to the propagation wavevector **k** direction of the evanescent wave. Therefore, for an arbitrarily polarized optical field, a three-dimensional SAM density can be obtained. Research on the three-dimensional SAM density, including transverse spin components, has mainly focused on evanescent wave systems, including evanescent waves generated by total internal reflection and surface plasmon polaritons. Notably, research related to the SAM of surface plasmon polaritons has received considerable attention in recent years, leading to interesting research hotspots such as photonic skyrmions, optical spin lattice structures, and plasmonic optical tweezers [152,153].

**Figure 4:**
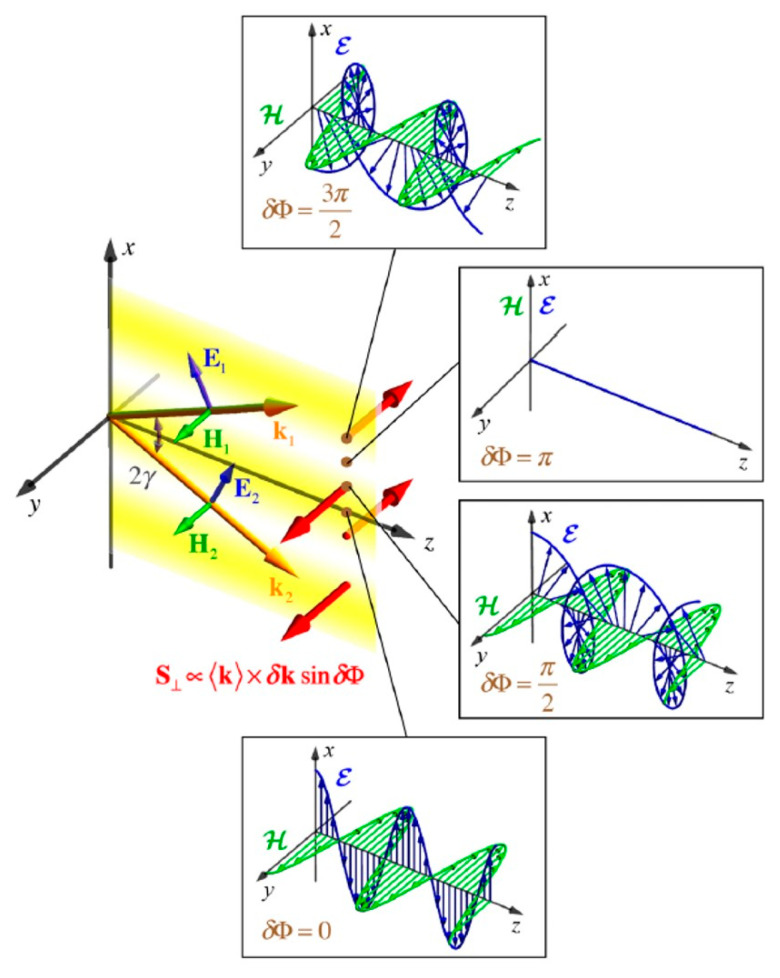
The interference of two linearly polarized propagating plane waves leads to the generation of t-SAM density. The illustrations correspond to the changes in electric and magnetic fields along the *z*-axis at different *x* positions [12].

**Transverse Spin in Interference Fields:**

t-SAM does not only exist in evanescent fields but can also be generated in free space under certain conditions, such as in interference fields and tightly focused fields. Below, a simple two-wave interference configuration is discussed [154]. Assume two beams of light propagating along the *z*-direction, as shown in Figure 4, with the same amplitude and an interference angle of 2*γ* between them in the *x*-*z* plane. Their respective amplitude forms are

(11) $$\begin{array}{l} E_{1,2}=\frac{A_{0}}{\sqrt{1+{\left|m_{1,2}\right|}^{2}}}(\frac{k_{z}}{k}\bar{x}+m_{1,2}\bar{y}\mp \frac{k_{x}}{k}\bar{z})\exp(i\Phi_{1,2}),\\ H_{1,2}=\frac{k_{1,2}}{k}\times E_{1,2},\Phi_{1,2}=k_{z}z\pm k_{x}x \end{array}$$

The interference result is the superposition of the complex amplitudes of the two beams, $E=E_{1}+E_{2},H=H_{1}+H_{2}$. Therefore, the total interference field is expressed as

(12) $$E=\frac{2A_{0}}{\sqrt{1+{\left|m_{1,2}\right|}^{2}}}(\frac{k_{z}}{k}\cos\frac{\delta \Phi }{2}\bar{x}+m\cos\frac{\delta \Phi }{2}\bar{y}-i\frac{k_{x}}{k}\sin\frac{\delta \Phi }{2}\bar{z})\exp(ik_{z}z)$$

where $\delta \Phi =\Phi_{1}-\Phi_{2}=2k_{x}\Delta x$ represents the phase difference between the two beams in the x direction, and the polarization states of the two beams are the same: $m_{1}=m_{2}=m$, $\left(\tau_{1},\chi_{1},\sigma_{1})=(\tau_{2},\chi_{2},\sigma_{2})=(\tau ,\chi ,\sigma \right)$. Note that the longitudinal electric field component $E_{z}\propto -ik_{z}\sin(\delta \Phi /2)k\bar{z}$. By analogy with the $E_{z}$ component in evanescent fields, both are imaginary components. $E_{z}$ differs from the transverse electric field components by an imaginary unit i, meaning that for the interference field, the field quantities can also rotate in the *x*-*z*, *y*-*z* planes, generating a transverse SAM density perpendicular to the total wavevector **k**.

It can be found that the t-SAM density here is generated by the electric field components. However, light has both electric and magnetic components, so the t-SAM density caused by both electric and magnetic parts can be obtained. Taking the interference of two beams with arbitrary polarization states as an example, using Equations (4) and (12), the SAM density including both electric and magnetic parts is

(13) $$S^{e,m}\propto {\left|A_{0}\right|}^{2}\left[\sigma \frac{k_{z}}{k}(1+\cos\delta \Phi )\bar{z}+(1\pm \tau )\frac{k_{x}k_{z}}{k^{2}}\sin\delta \Phi \bar{y}\mp \chi \frac{k_{x}}{k}\sin\delta \Phi \bar{x}\right]$$

It can be seen that the SAM density generated by the interference of arbitrarily polarized beams consists of three parts: the l-SAM density $S_{z}$ related to the polarization ellipticity σ; the t-SAM density related to τ; and the t-SAM density $S_{x}$ related to χ. The fundamental reason for the generation of these SAM densities is that interference causes $E_{z}$ to have an imaginary part, leading to the rotation of field components in the *x*-*z* or *y*-*z* planes. This is similar to the case of evanescent fields, but there are differences. In the interference field, the t-SAM densities $S_{y}$ and $S_{x}$ are both related to sinδΦ. Noting that $\delta \Phi =\Phi_{1}-\Phi_{2}=2k_{x}\Delta x$, therefore, $S_{y}$ and $S_{x}$ are not uniformly distributed in space but oscillate sinusoidally in the *x* direction, whereas for an evanescent plane wave, it is uniform and constant.

### 2.3. Spin-Momentum Locking Relationship

The decomposition of photon longitudinal spin and transverse spin mentioned above is distinguished by the wavevector **k**. The component of SAM perpendicular to the wavevector **k** direction is called transverse spin, and the component parallel to it is called longitudinal spin. This empirical approach provides an intuitive method for identifying SAM. However, the method of distinguishing by the wavevector **k** is not applicable in some complex structured fields, such as when considering structured optical fields with arbitrary trajectories and OAM. Considering the relationship $p^{s}=(\nabla \times S)/2$ between the SAM density and the spin-momentum density in vector optical fields, this provides an idea for quantitatively describing the SAM density in structured optical fields.

Shi et al. proposed a set of spin-momentum equations, analogous to Maxwell’s equations, to describe the dynamic changes in SAM density and momentum density. Simultaneously, this set of equations generalizes the property of optical spin-momentum locking from evanescent plane waves to general evanescent fields [15,16,17].

For any electromagnetic wave propagating in a homogeneous, non-dispersive medium, the curl of the Poynting momentum density can be expressed as

(14) ∇×P=1εμ∇×p=ω2S−14Re−∇⊗E*·H−∇⊗EΤ·H*+∇⊗H*·E+∇⊗HΤ·E*

Here, ⊗ denotes the following operation:

(15) $$r_{1}⊗r_{2}=\left(\begin{array}{ccc} {x_{i}}^{1}{x_{i}}^{2} & {x_{j}}^{1}{x_{i}}^{2} & {x_{k}}^{1}{x_{i}}^{2}\\ {x_{i}}^{1}{x_{j}}^{2} & {x_{j}}^{1}{x_{j}}^{2} & {x_{k}}^{1}{x_{j}}^{2}\\ {x_{i}}^{1}{x_{k}}^{2} & {x_{j}}^{1}{x_{k}}^{2} & {x_{k}}^{1}{x_{k}}^{2} \end{array}\right)$$

From Equation (14), it can be seen that there is a correlation between the SAM density **S** and the vorticity of the Poynting momentum **p**, which manifests as an intrinsic spin-momentum locking of light. The additional term is similar to a term generating a geometric phase [155]. Further shifting the object of discussion from homogeneous propagating fields to evanescent fields possessing t-SAM density, Equation (14) can be simplified to

(16) $$S=\frac{1}{2\omega^{2}}\nabla \times P=\frac{1}{2k^{2}}\nabla \times p$$

Here, the SAM density **S** is proportional to the curl of the Poynting momentum **p**. This reveals that the SAM originates from the inhomogeneity of the energy flow, manifested as the vorticity of the energy flow. For the case of evanescent fields, the previous discussion indicates that the electromagnetic field decays along the interface normal, which is a manifestation of energy flow inhomogeneity, thus capable of generating a t-SAM density perpendicular to the propagation wavevector k. It is important to note that this equation cannot yield l-SAM, such as that of a circularly polarized plane wave where the Poynting momentum is uniform in spatial distribution. Additionally, this equation describes the spin-momentum locking property of evanescent fields: the transverse spin vector varies around the Poynting momentum density from an “up” to a “down” state, forming a chiral vortex structure of the 2D transverse spin. This chiral structure is locked to the energy propagation direction and satisfies a right-hand rule. That is, if the energy flow density reverses from forward (+P) to backward (−P), the direction of the local transverse spin vector will flip. The spin-momentum locking relationship is also significant for the detection of SAM; it allows for inferring the transverse SAM by measuring the energy flow, or vice versa.

Recently, Shi et al. extended this spin-momentum locking property to general optical fields and several special classical fields [17], including longitudinal acoustic waves and surface water waves (as shown in Table 1), finding consistent properties.

The kinetic momentum and properties of water and acoustic waves as well as those of linearly polarized surface EM waves can be found in Ref. [17]. For the longitudinal acoustic wave, $c_{A}^{2}=1/\beta_{A}\rho_{A}$ is the speed of the acoustic wave, where $\beta_{A}$ is the compressibility of the acoustic medium; $\rho_{A}$ is the mass density of the acoustic medium; $\omega_{A}$ and $k_{A}=\omega_{A}/c_{A}$ are the angular frequency and wavenumber, respectively. Similar relations apply to gravity water waves; the $\sigma_{G}$ and $\sigma_{A}$ denote the chirality parameters of gravity water waves and acoustic waves, respectively. Since their field-theoretical descriptions correspond to spin-0 scalar fields, their intrinsic chirality is zero.

### 2.4. Topological Properties of Optical Transverse Spin

**Figure 5:**
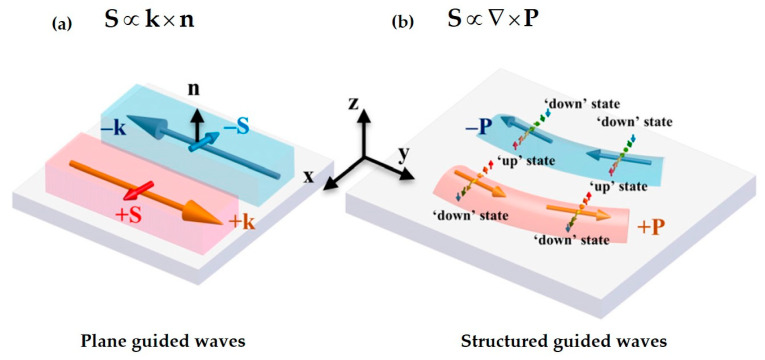
The spin-momentum locking properties of structural evanescent fields [15]: (**a**) in evanescent plane wave, optical spin-momentum locking results in the transverse spin **S** uniformly distributed and parallel to the interface. The spin vector direction is perpendicular to the wavevector **k** and flips if the propagation direction flipped from +**k** to −**k**; (**b**) in the structural evanescent field, the optical spin is related to the vorticity of the energy flow density **P**/Poynting momentum **p**. The transverse spin vector varies from the “up” state to the “down” state around the energy flow density, remaining perpendicular to the local wavevector. This forms a chiral swirl of the 2D transverse spin which is locked to the energy propagating direction and fulfills a right-handed rule. The direction of the local transverse spin vector flips if the energy flow density flipped from forward (+**P**) to backward (−**P**).

In addition to uncovering the physical dynamics of transverse spin, Equation (16) indicates a unique spin-momentum locking feature associated with this spin. For the evanescent plane wave at a vacuum/metal interface, oppositely propagating evanescent waves with +**p** and −**p** along the *y*-direction carry opposite transverse optical spins $S_{x}$ > 0 and $S_{x}$ < 0 along the *x*-direction, with the direction of the t-SAM locked to the kinetic momentum of the wave. This universal feature of spin–momentum locking is considered a photonic analog to the quantum spin Hall effect of electrons in topological insulators [80,156,157,158,159]. This feature underlies the spin-controlled directional coupling of evanescent waves such as SPPs. For structured evanescent waves with spatially varying intensity distributions, inhomogeneity in the momentum density induces both in-plane and out-of-plane t-SAM components introduced above. Both are perpendicular to the local direction of momentum. The relationship between the two components leads to a chiral spin texture with spin vectors swirling around the momentum lobe (Figure 5a,b), its directional variation (i.e., chirality) being locked with the momentum. The orientation of the spin vectors varies progressively from the ‘up’ state to the ‘down’ state across the momentum lobes, while obeying the right-hand rule (at the vacuum half space). This progression is a manifestation of a generalized spin–momentum locking associated with an arbitrary structured evanescent wave.

It is established that the total Chern number of a surface mode vanishes (*C_t_* = 0), a consequence of the time-reversal symmetry inherent in non-magnetic Maxwell’s equations [15,24,78]. A Chern number is a topological invariant that quantifies the topological properties of a system, for example, the number of chiral states. It is calculated as the integral of the Berry curvature over the space. Here, the Chern number is related to the symmetry of optical system, and a non-zero Chern number indicates a nontrivial topological phase, distinguishing it from a “trivial” phase. In contrast, the spin Chern number is non-zero (*C_spin_* = 4). This non-zero value confirms the existence of nontrivial helical states of electromagnetic waves, which are strictly locked to the direction of energy propagation [15]. Although the existence of such nontrivial helical states at the interface governed by the spin-momentum locking, the topological $Z_{2}$ invariant of these states vanishes owing to the time-symmetry of Maxwell’s equations. Consequently, the optical transverse spin-momentum locking discussed herein is distinct from the “pseudo-spin” engineered in artificial photonic structures [80,158,159]. The latter is designed to break time-reversal symmetry and thus enjoys protection against back-scattering. While the interconversion between the two helical states of evanescent waves lacks topological protection against scattering, the spin-momentum locking itself—along with the resulting unidirectional excitation and transport of photons—constitutes an intrinsic feature of Maxwell’s theory and is topologically nontrivial, characterized by a topological $Z_{4}$ invariant [15,24,160].

**Figure 6:**
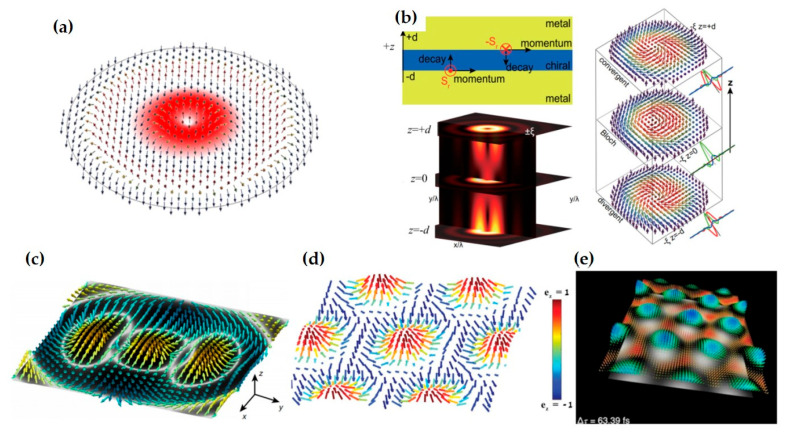
Various kinds of photonic topological structures in guided waves formed in real space: (**a**) Néel-type photonic spin skyrmion formed in an evanescent optical vortex, for which the spin vector varies progressively from the ‘up/down’ state at the center to the ‘down/up’ state at the edge (with integer skyrmion number) [161]; (**b**) Bloch-type photonic spin skyrmion in guided waves introduced into a layer of a chiral material which induces an **E**–**H** coupling [25]; (**c**) meron-like photonic spin structure in a specially designed SPP field [88]; (**d**,**e**) lattices exhibiting 6-fold rotational symmetry of dynamic field skyrmions produced by an evanescent field in the absence spin–orbit coupling, mapped (**d**) spatially [163] and (**e**) temporally [164].

Notably, the spin vector aligns tangentially to the interface at the momentum density maxima and normal to it at the nodes. This allows the spin variation period to be defined between two adjacent nodes of the kinetic momentum density, a behavior exhibited by topological solitons [83,84,161,162]. Consequently, spin–orbit coupling gives rise to a variety of intriguing photonic spin structures. Examples include the Néel-type photonic spin skyrmion in an evanescent optical vortex (Figure 6a) [24,84,85,161], the Bloch-type skyrmion emerging with a layer of chiral material (Figure 6b) [24,25,85,161], and meron-like spin structures in specially designed fields (Figure 6c) [88]. In the absence of spin–orbit coupling, however, these spin topologies degenerate into dynamic field–skyrmions (Figure 6d,e) [163,164]. These compelling real-space topological structures and their ultrafast dynamics have garnered significant recent interest, opening new pathways in topological photonics, quantum photonics, metrology, and optical manipulations [165], while also providing fresh avenues for exploring topological condensed matter systems [155,166,167].

**Figure 7:**
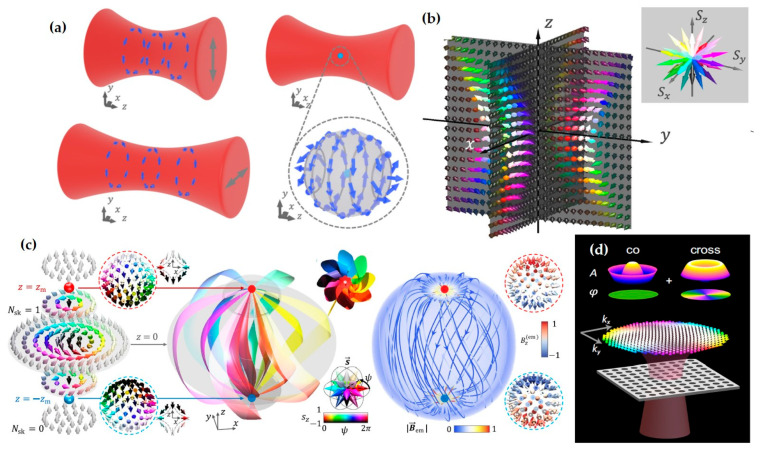
Various kinds of photonic spin topological structures in free space: (**a**) a topological spin defect at the focal point and the corresponding normalized spin vectors [169]; (**b**) photonic spin Hopfions in 3D real space [170]; (**c**) a toron topology in high-dimensional space [171]; (**d**) the generation of meron topologies in BICs [172].

The aforementioned topological solitons are two-dimensional, which emerge at the optical near-field. Whereas in the free space, high-dimensional topologies can be constructed. For example, Wang et al. discovered a type of topological spin defect in 3D space is associated with a point where the electromagnetic spin density is zero [168], as shown in Figure 7a. It is generical that there is a nontrivial topological spin texture surrounding the null point, and thus a topological spin defect possesses a quantized topological charge. They provide examples of diverse defects in 3D space, such as isolated defect points, periodic or quasi-periodic defect lattices. Then, Wang et al. structured the monochromatic light to make the photonic spin exhibit a hopfion texture in the three-dimensional real space [169], as shown in Figure 7b. They provided ways to construct spin textures of arbitrary Hopf charges. And then, they extended the system to four dimensions by introducing an additional parameter dimension and constructed a new type of topological defect in the form of a monopole loop carrying quantized Hopf charges, in which each point on the loop is a topological spin defect in three dimensions. Very recently, Shen et al. constructed torons [170], as shown in Figure 7c, which are a class of 3D chiral topological textures with both skyrmionic quasiparticle textures and monopole point defects with the photonic spin, and demonstrate the topological phase transitions among different 3D topological states: torons, hopfions, skyrmioniums, and monopole pairs. In addition, the toron’s chirality and monopole’s helicity can be continually tuned. In addition, the topological defects of photonic spin can be present in bound states in the continuum (BICs) [171], as shown in Figure 7d. Under a circularly polarized light illumination, the vortex topology of the momentum space of BICs can transform light with spin textures of meron topology in momentum space. The topologies exhibit polarity-switchable configurations, which can be controlled by the polarization of incident light. In conclusion, the high-dimensional topological textures and the associated topological phase transitions are prevailing in free space [172,173,174,175], which is potential in control and sensing of nanoparticles, and optical generation of topological texture in motions of particles or fluids.

## 3. Detection Methods for Spin Angular Momentum

As an intrinsic property of the optical field itself, SAM can exist in various systems such as paraxial beams, tightly focused fields, interference fields, and evanescent fields. The corresponding detection methods also vary, but based on the locality of the optical field, they can basically be divided into two categories: far-field and near-field. The former mainly involves paraxial beams and focused beams. Generally, these two types of optical fields are considered as scalar fields; therefore, the SAM only has a component parallel to the propagation wavevector **k**. Thus, extracting the left-handed or right-handed circularly polarized component can detect the SAM. The latter generally refers to evanescent fields and the interference of evanescent fields. These optical fields are considered vector fields localized in the optical near-field. Additionally, the detection of different field components in tightly focused fields also relies on near-field methods for independent extraction. Therefore, the detection of SAM mainly concerns the near-field situation. To address this issue, researchers have proposed several types of detection techniques, primarily based on scanning near-field optical microscopy, nanoparticle-on-film structures, photoemission electron microscopy, and detection methods based on nonlinear effects. These methods can effectively extract different electromagnetic field components, thereby obtaining information about the SAM carried by the optical field.

### 3.1. Spin Angular Momentum Detection Based on Near-Field Scanning Optical Microscopy

Near-field scanning optical microscopy is an important means for optical near-field detection. The initial purpose of this technology was actually to break the diffraction limit for imaging, first proposed by British scientist E. H. Synge in 1928 [176]. With technological advancements, researchers further achieved the extraction of different electromagnetic field polarization components, which is crucial for obtaining information about the SAM of the optical field.

**Figure 8:**
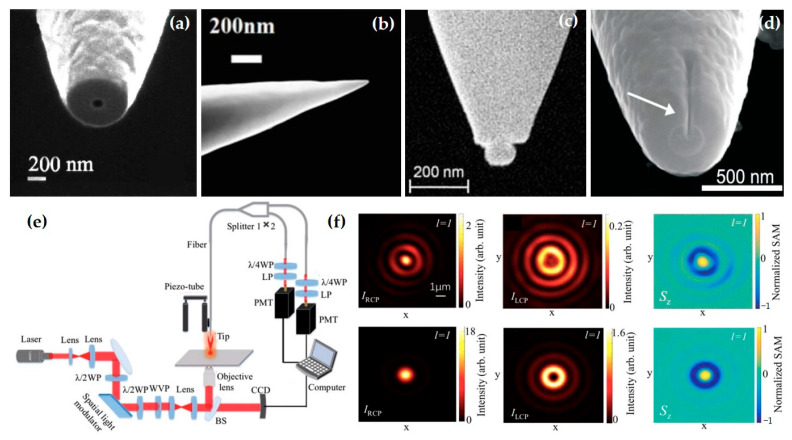
Schematic diagram of several typical near-field optical probes and a SAM detection system based on NSOM: (**a**) aperture-type probes formed by cutting off the top of the metal coating on optical fibers using a focused particle beam [178]; (**b**) scattering-type near-field probe [179]; (**c**) cone-shaped glass probe with gold nanoparticles attached to the tip [13]; (**d**) side wall aperture-type probe for measuring near-field optical frequency components [180]; (**e**) the experimental optical setup for detecting the near-field SAM distribution using the NSOM system, one quarter-wave plate and linear polarizer are used to extract the left and right circularly biased components. (**f**) The experimental results obtained from the experimental optical path shown in Figure (**e**), the configuration of the incident beam is a radial first-order vortex beam [103].

The detection in NSOM systems is primarily achieved by designing optical probes. The type and size of the optical probe simultaneously determine the resolution of near-field characterization and the category of characterized electromagnetic components. Based on whether the probe tip has an aperture, NSOM systems are divided into two types: one is the aperture-based NSOM (a-NSOM), where the probe tip has an aperture. This type of probe is often used for extracting in-plane components ($E_{x}$ and $E_{y}$) (Figure 8a). The other is the scattering-type NSOM (s-NSOM), where the probe tip has no aperture. This type of probe is often used for measuring one longitudinal component ($E_{z}$) and one in-plane component, Topological Magnetic Lattices for On-Chip Nanoparticle Trapping and Sorting ($E_{x}$ and $E_{y}$). Because its size is smaller than a-NSOM probes, its resolution is also higher, reaching several nanometers (Figure 8b). When considering the electromagnetic response of the probe, the probe tip is generally treated as a dipole, which converts the near-field electromagnetic signal to the far-field. For scattering-type probes, the interaction between the probe and the near-field electric field generates an electric dipole moment **p**, where **p** and the near-field **E** satisfy p=α·E. Here, α is the polarizability tensor, representing the response relationship between the near-field signal and the far-field signal. Aperture-type probes additionally require obtaining the polarization transmission matrix of the probe acting as a waveguide.

To detect or enhance specific electromagnetic field components, researchers have further designed the shape of the probe tip based on the two types of probes. As shown in Figure 8c, placing a nanoparticle on the end face of the probe enables the extraction of both in-plane and out-of-plane electric field components [100,101]. Clearly, this is significant for extracting both the transverse and longitudinal components of SAM. Besides electric field components, the acquisition of magnetic field vectors cannot be ignored. Taking the SAM generated in surface plasmon polariton systems as an example, the magnetic component also contributes to the generation of t-SAM. To detect magnetic field information, researchers introduced a horizontal magnetic dipole using a hollow pyramidal aperture probe (Figure 8d), thereby achieving the extraction of transverse magnetic field components [177]. Using specially designed near-field probes, researchers have detected SAM. In 2020, Yin et al. from Shenzhen University proposed a tapered aperture probe. By drilling a hole in the tip of a gold-coated fiber probe, they achieved the acquisition of in-plane electric field components. Using a combination of a quarter-wave plate and a linear polarizer, they obtained the near-field circular polarization signal, ultimately achieving detection of the l-SAM $S_{z}$, as shown in Figure 8e,f.

### 3.2. Near-Field Coupling Resonance Effect of Nanoparticle-on-Film Structures

Traditional NSOM systems can respond to different electromagnetic field quantities by designing different types of probes, thereby obtaining information about the SAM in the optical near-field. However, this technique is limited by the complexity of the system itself and the cost of fabricating and using nanometer probes, restricting its application in near-field SAM detection. To address these issues, researchers, starting from the dipole radiation effect of near-field probes, introduced nanoparticles as near-field probes. Particles in the near-field can also produce a dipole radiation effect, equivalent to the probe tip in an s-NSOM system. Placing a nanoparticle on a metal film enables imaging of the surface plasmon polariton distribution at the metal–air interface. Additionally, this nanoparticle-on-film (NP-on-film) structure is also commonly used for extracting field components of focused fields. Its composition is simpler compared to general NSOM systems, and specially designed probe particles can be more flexibly used for detecting different field components.

**Figure 9:**
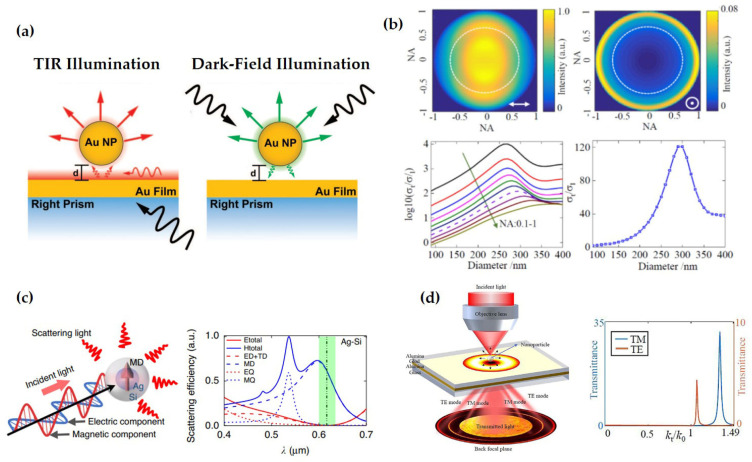
Nanoparticle–film structures for the detection of SAM: (**a**) two different excitation methods of nanogold particle–gold film structure [181]; (**b**) research on optical response of PS nanoparticles [102]; (**c**) Ag core–Si shell probe particles used for detecting magnetic field components, the electromagnetic response curve of the particles is shown in the figure on the right [91]; (**d**) schematic diagram of the coupling process between tightly focused beam and nanoparticle–waveguide structure. The right figure shows the calculation of Fresnel coefficients for different modes in this structure [104].

Depending on the characterized optical field, two different types of NP-on-film structures are typically designed: one is the nanoparticle–metal film structure for evanescent field detection, and the other is the nanoparticle–waveguide structure for focused field detection. These two structures correspond to two different excitation methods [181], as shown in Figure 9a. The nanoparticle–metal film structure corresponds to the total internal reflection (TIR) illumination method, where incident light first excites surface plasmon polaritons (SPPs) on the metal film surface, which then excite the scattered light and localized surface plasmons (LSPs) of the metal particle. In contrast, the nanoparticle–waveguide structure corresponds to the dark-field illumination method, where incident light first illuminates the metal particle, exciting the particle’s scattered light and LSPs, and then the scattered light excites SPPs on the metal film surface.

Using the nanoparticle–metal film structure, scanning imaging of the spin components of the surface plasmon wave field can be achieved. For example, Li et al. used focused vector beams to excite SPP fields and discovered localized spin structures—photonic skyrmions—caused by spin–orbit coupling [84]. The experimental setup is as follows: an oil immersion objective excites SPPs at the silver film–air interface; a 300 nm diameter PS particle radiates the near-field electric field components to the far-field; and an objective with NA = 0.7 collects and extracts the left-handed and right-handed circularly polarized components. It is important to note that the NA = 0.7 objective primarily collects in-plane components because the in-plane electric field components excite dipoles oscillating in the plane on the particle, whose radiated electric field is distributed within a small numerical aperture range, while the out-of-plane electric field components are distributed at large numerical aperture angles. Therefore, the in-plane electric field components $E_{LCP}$ and $E_{RCP}$ can be obtained, and subsequently, the SAM component $S_{z}$ parallel to the SPP wavevector ($S_{z}\propto (I_{RCP}-I_{LCP})$) can be derived. Furthermore, based on the nanoparticle–metal film structure, researchers have also discovered various spin topological structures, such as optical meron lattices and skyrmion lattices. These optical spin topological structures have application potential in nanometrology, data storage, super-resolution imaging, and chiral detection. Besides electric field components, the optical SAM is also related to magnetic components. To detect magnetic components, Meng et al. from Shenzhen University designed the probe particle and proposed a core–shell structured nanoprobe [91]. This probe consists of a silver metal core and a silicon dielectric shell, behaving as a pure magnetic dipole with no electric field response. Using this probe, they achieved for the first time the measurement of magnetic topological spin structures, including individual skyrmions and meron/skyrmion lattices.

The main difference between the nanoparticle–waveguide structure and the nanoparticle–film structure is that the waveguide part of the former is composed of a dielectric–metal–dielectric multilayer film, involving different electromagnetic mode interactions [104]. Yang et al. from Shenzhen University first proposed using this structure to detect the three orthogonal polarization components in tightly focused optical fields [104]. The basic principle of their design is that the interaction between the nanoparticle and the focused optical field generates scattered light with multiple wave vectors. Part of the scattered light, satisfying the resonant excitation condition of the designed waveguide, can be coupled by the waveguide and radiated to the far-field. Based on the different resonance angles, different resonance modes can be separated, including pure TM modes, pure TE modes, and mixed modes of the two. Using the separated TM and TE modes, the three components of the SAM can be obtained. The relationship between the SAM components and the electromagnetic field modes was first given by Peter Banzer et al. in 2015 [94].

### 3.3. Spin Angular Momentum Detection Based on Nonlinear Effects

Both the detection methods based on NSOM systems and nanoparticle–film structures have certain limitations because obtaining the SAM distribution relies on probe scanning, which is affected by the displacement accuracy and scanning time of the detection system. Therefore, a detection method that does not rely on probe scanning and allows real-time imaging is needed. In 2021, Kobi Frischwasser et al. proposed a nonlinear near-field optical microscopy (NNOM) system, which can achieve real-time imaging of evanescent waves through nonlinear effects [99].

**Figure 10:**
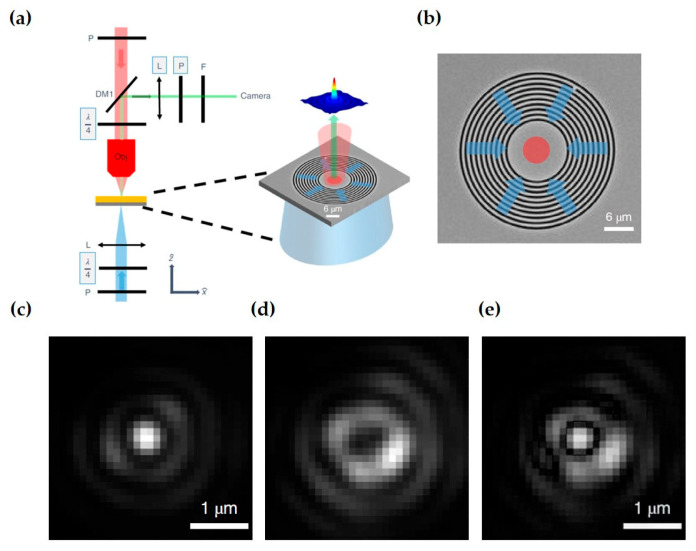
SAM detection system based on nonlinear effects: (**a**) optical path configuration of nonlinear near-field optical microscopy [99]; (**b**) circular coupled grating structure for exciting SPP [99]; (**c**,**d**) represent the near-field distribution obtained by illuminating the grating with left-handed and right-handed circularly polarized pump beams, respectively; (**e**) shows the author’s differential results on the two figures.

This imaging method using NSOM is mainly based on the nonlinear four-wave mixing effect, converting the spatial and temporal information carried by SPPs into detectable optical signals in the far-field. It should be noted that, besides four-wave mixing, other nonlinear effects could theoretically also achieve this function. Below, using the work of Kobi Frischwasser et al. as an example, this detection method based on nonlinear effects is introduced. The condition for generating nonlinear signals that can radiate to the far-field is satisfying energy conservation and in-plane momentum conservation, i.e., $\omega_{n1}=2\omega_{pump}-\omega_{SPP}$,$k_{n1,∥}=2k_{pump,∥}-k_{SPP,∥}^{*}=-k_{SPP,∥}^{*}$. From the expression, it can be seen that the far-field propagating nonlinear signal is related to the pump light and SPPs. By controlling the pump light, different in-plane signals can be extracted. As shown in Figure 10a, the blue, red, and green beams represent the excitation light ($\lambda_{exc}=1030\;\mathrm{nm}$), the pump light ($\lambda_{pump}=800\;\mathrm{nm}$), and the generated nonlinear signal ($\lambda_{nl}=654\;\mathrm{nm}$), respectively. Circularly polarized excitation light is incident on a circular metal grating to generate surface evanescent waves (SPPs). The pump light is used to generate radiatable nonlinear signals. These signals are then separated from the pump beam by a dichroic mirror. Changing the fast axis direction of the quarter-wave plate allows extraction of different in-plane components. Figure 10b shows the extracted in-plane right-handed and left-handed circularly polarized signals. Additionally, this work also provided a video showing the transition of the extracted signal from left-handed to right-handed circular polarization, demonstrating the system’s advantage in real-time measurement. Figure 10c–e shows the difference result of the extracted left-handed and right-handed circularly polarized signals, which can reflect the l-SAM of the SPP field.

### 3.4. Spin Angular Momentum Detection Using Photoemission Electron Microscopy

With the development of ultrafast optics, researchers have conducted in-depth studies on the evolution of electromagnetic fields at extremely small spatial and temporal scales. As one of the degrees of freedom of light, the SAM constitutes special spin structures whose spatial distribution, in theory, does not change with the oscillation of electromagnetic field components. However, in research on light–matter interactions, nonlinear dynamics, and the manipulation of optical spin structures, the processes and interactions involving optical spin structures have dynamic characteristics. Therefore, detecting optical spin structures on ultrafast timescales is of great significance.

Different from general near-field spin angular momentum detection methods, time-resolved photoemission electron microscopy (TR-PEEM) has the advantage of being able to image the ultrafast dynamics of coherent electronic excitations (plasmons, excitons, etc.) in solid-state materials. Multi-photon photoemission (MPP) can be excited using degenerate (same wavelength and intensity) or two-color pump-probe excitation schemes, where the first pulse generates the excitation and the second pulse probes its spatial and temporal evolution by inducing photoemission from the excited state. TR-PEEM technology is particularly suitable for measuring plasmonic phenomena on nano–metal structure surfaces because it can image plasmonic wave fields with subwavelength resolution, and the interference between the plasmonic wave field and the external field provides strong contrast.

**Figure 11:**
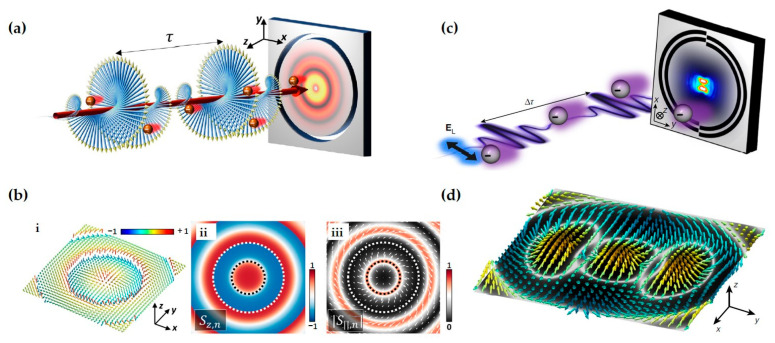
SAM detection system based on PEEM: (**a**) schematic diagram of the coupling between circularly polarized light and a grating [86]; (**b**) three corresponding vectorial diagrams of the photonic skyrmion structure generated by the coupling between circularly polarized light and the grating, showing the longitudinal component $S_{z}$ and the in-plane component $S_{∥}$ of the skyrmion structure; (**c**) schematic of the coupling of linearly polarized light to an Archimedean helix grating [88]; (**d**) vector distribution of the spin meron [88].

In research on optical spin structures using photoemission electron microscopy, Dai et al. used interferometric time-resolved two-photon photoemission electron microscopy (ITR-2P-PEEM) to measure and verify the existence and distribution of photonic spin skyrmions, recording a series of PEEM images (Figure 11a,b) of the plasmonic vortex excited by circularly polarized light at the center of a circular coupling structure [86]. By coupling 550nm, 20fs circularly polarized light with a circular grating etched on a Ag film, they generated photonic skyrmions and simultaneously performed ultrafast imaging of the plasmonic vortex field.

With PEEM, Dai et al. also discovered a topological meron structure [88]. This spin structure was generated by superimposing two plasmonic vortex fields. Experimentally, a linearly polarized beam was used to illuminate a silver film etched with an Archimedean spiral structure (Figure 11c), exciting surface plasmons with a structural topological charge m=2. Since linearly polarized light is a superposition of left and right circularly polarized light, carrying SAM of σℏ=±ℏ, it generated two plasmonic vortices with a total topological l=m+σ, forming a spin half-integar structure (Figure 11d).

## 4. Application Research of Spin Angular Momentum

### 4.1. Weak Effect Measurement

The photonic spin Hall effect (SHE) is an important phenomenon in optics, describing a tiny transverse shift experienced by a light beam during propagation due to differences in its polarization state (spin). This effect reveals the microscopic dynamics of light–matter interaction and is significant for precise measurement and detection of various physical quantities, especially in the field of weak effect detection.

Leveraging the high sensitivity of the SHE to changes in photoconductivity in media, the team of Wen at Hunan University used the SHE as a pointer for precisely measuring the photoconductivity of graphene, making important progress in weak effect measurement [35]. In related work, Wen et al. measured the photoconductivity of monolayer graphene as $(0.993\pm 0.005)\sigma_{0}$, achieved a higher measurement resolution of $1.5\times 10^{-8}\Omega^{-1}$, and found that for untwisted few-layer graphene, the photoconductivity increases linearly with the number of material layers. Furthermore, this weak measurement amplification technique based on the SHE of light holds promise for measuring other parameters of atomically thin crystals, such as magneto-optical constants, circular dichroism, and optical nonlinear coefficients.

**Figure 12:**
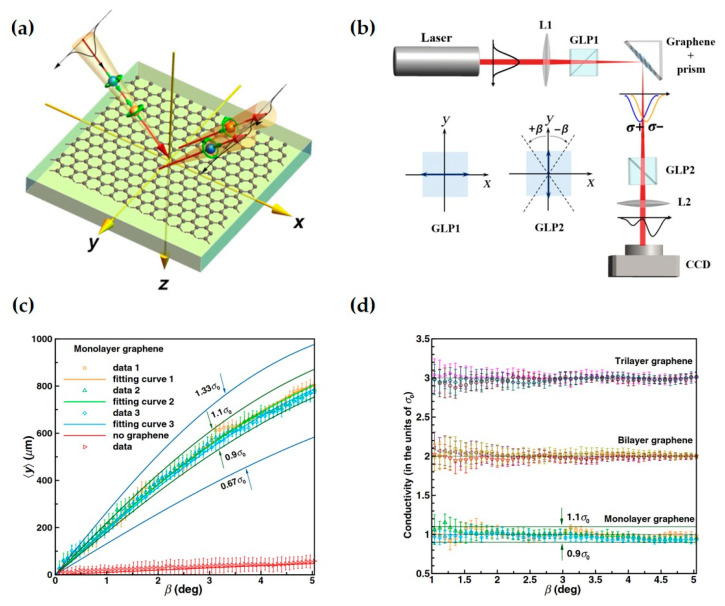
Precision measurement of graphene’s optical conductivity by SHE [35]: (**a**) SHE at an air-graphene interface; (**b**) experimental setup to detect the tiny spin–orbit interaction of light in graphene; (**c**) amplification of pointer shifts as a function of the post-selected angle β; (**d**) measurement of the optical conductivity in the unit of $\sigma_{0}$ for monolayer, bilayer, and trilayer graphene.

As shown in Figure 12a, when a light beam is incident on a graphene interface, the SHE occurs, causing the two spin components to separate at a very small angle, forming a spin-dependent displacement. This displacement is determined by the Fresnel coefficients and is related to the photoconductivity. Therefore, using weak measurement techniques, this spin-dependent displacement can be used as the pointer. By measuring the change in the pointer, the photoconductivity of the atomically thin crystal can be obtained.

### 4.2. Optical Differentiation

Compared to traditional information processing methods, optical information processing offers advantages such as ultra-high speed, parallel processing, immunity to electromagnetic interference, and low energy consumption. A typical optical information processing method is optical differentiation, a technique that uses the difference between two or more optical signals for measurement and analysis.

The application of SAM in optical differentiation is also based on the SHE. SHE can cause a beam to undergo a tiny displacement during propagation due to different spin states. This displacement can be used for differential measurement of signals, greatly improving measurement sensitivity and accuracy. Below, a brief optical spatial differentiator proposed by Luo et al. at Hunan University serves as an example to introduce the principle and advantages of optical differentiation [36]. They proposed an optical spatial differentiator consisting of two orthogonal polarizers and a tilted polarization interface. In this device, the geometric SHE generated at the tilted polarization interface causes a spin-dependent beam displacement. Spatial differentiation is achieved through the two orthogonal polarizers, which can also be used for one-dimensional edge detection. Compared to traditional computer-based image processing, this optical differentiator not only has the advantages of high speed and low energy consumption but also holds great potential in real-time, high-throughput, and ultrafast parallel image processing, with application prospects in fields such as autonomous driving, augmented reality, and microscopic imaging.

**Figure 13:**
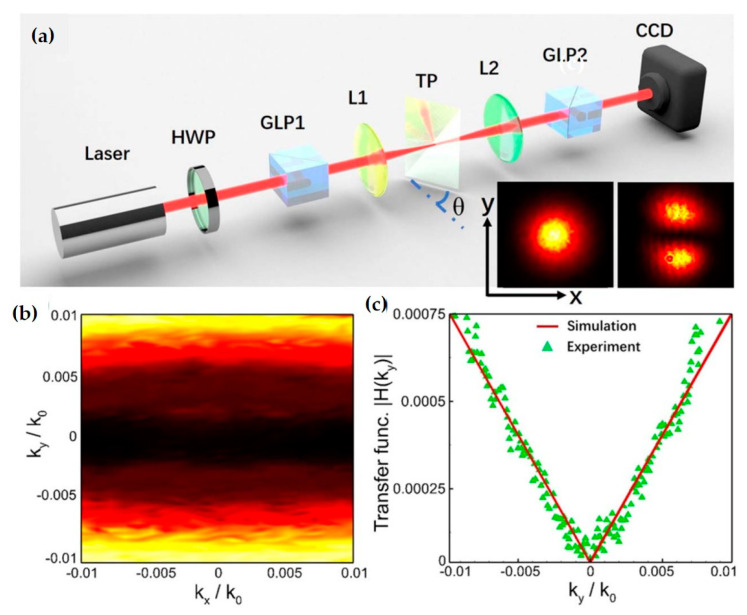
Spatial differentiation demonstration at a tilted polarizing interface [39]: (**a**) schematic of the experimental setup for observing splitting beams based on quantum weak measurement. The insets correspond to spatial differentiation results for a Gaussian illumination with the inclination angle *θ* = 45°; (**b**) measurement of the spatial spectral transfer function at a tilted polarizing interface; (**c**) detailed dates of the transfer function extracted from (**b**), when $k_{x}/k_{0}=0$.

Furthermore, Luo et al. found that spatial differentiation for optical computing can be achieved by analyzing specific orthogonal polarization states of light during reflection or refraction at a single optical planar interface [39]. It should be noted that spatial differentiation is essentially realized through the SHE. This effect usually accompanies the reflection and refraction of light at interfaces and is independent of material composition or incidence angle (Figure 13).

**Figure 14:**
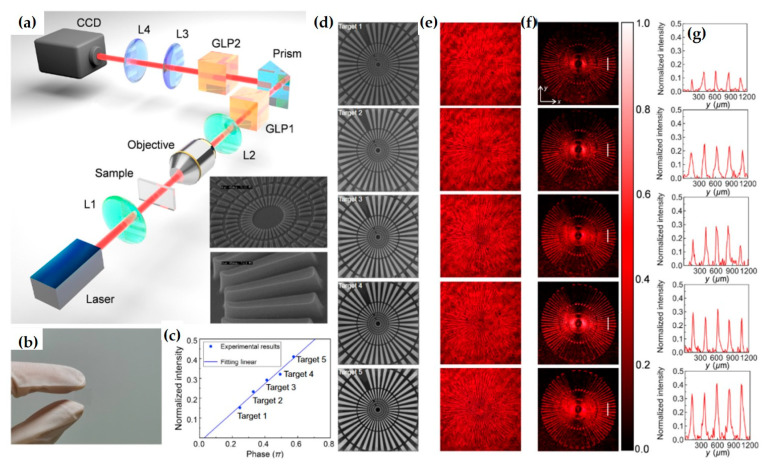
Microscopic imaging experiment on quantitative-phase-microscopy targets [40]: (**a**) experimental setup for photonic spin Hall differential microscopy. The insets in (**a**) are SEM images of a 350 nm focus star on the resolution target; (**b**) photograph of the phase target; (**c**) relationship between the horizontal-direction intensity of five edge images and the phase gradient of five focus-star targets; (**d**) images of targets 1–5 selected from user report for quantitative-phase-microscopy target. (**e**,**f**) Bright-field and differential images of the quantitative-phase target; (**g**) intensity curves corresponding to the white lines across the edge images in (**f**).

Further, since the SHE is related to the geometric phase and is not affected by wavelength, it can achieve broadband differential imaging of pure phase objects. Based on this characteristic, the group proposed a SHE microscopy technique (Figure 14) [40]. Its differential imaging of pure phase objects and unstained biological cells demonstrated excellent edge detection capability, offering potential for high-resolution reconstruction and quantitative analysis of phase images.

Compared to traditional differential interference contrast microscopes that require introducing complex phase contrast devices in bright-field microscopes, the SHE microscope requires only a simple glass interface, offering advantages of compact structure and low cost. This structure and characteristic give it application potential in multifunctional, high-contrast, real-time biological imaging.

**Figure 15:**
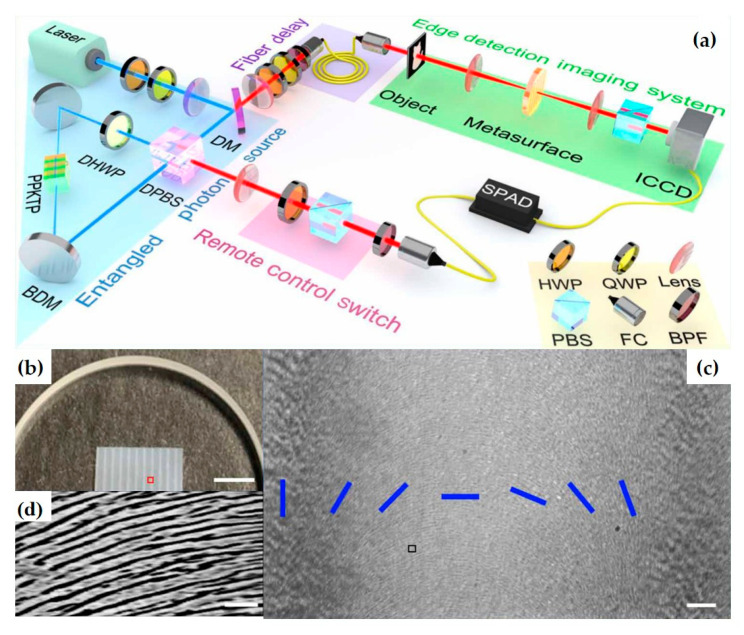
Experimental setup and sample characterization [47]: (**a**) experimental setup of metasurface-enabled quantum edge detection; (**b**) photograph of the partial metasurface sample. Scale bar, 4 mm; (**c**) polariscopic analysis characterized by crossed linear polarizers of the sample area marked in (**a**). The blue bars indicate the orientation of rotated nanostructures in one period, which represents the Pancharatnam–Berry phase induced by the laser writing dielectric metasurface. Scale bar 50 nm; (**d**) the scanning electron microscopy image of the sample area marked in (**c**).

The team also proposed an imaging system based on efficient dielectric metasurfaces, using a polarization-entangled photon source to turn the optical edge detection mode on or off [47]. This experiment enriches the fields of both metasurfaces and quantum optics, representing a promising direction for quantum edge detection and image processing with significant signal-to-noise ratio (Figure 15).

**Figure 16:**
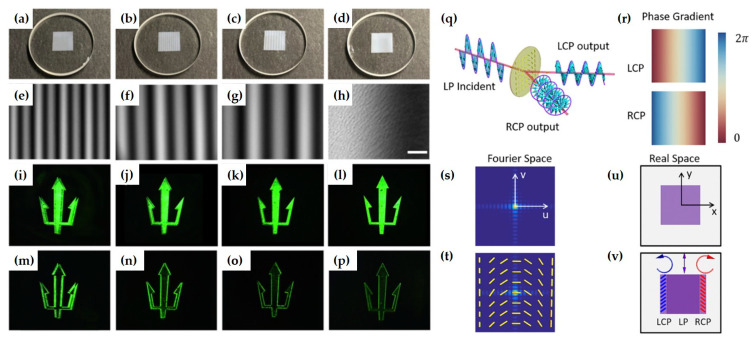
Experimental schematic of edge detection based on phase gradient metasurface [38]: (**a**–**p**) edge detection with various resolutions at the wavelength of 500 nm; (**q**–**v**) the proposed concept of edge detection based on a designed phase gradient metasurface. (**q**) When PB phase gradient metasurface is illuminated by collimated LP beam, two separated LCP and RCP beam components are observed. (**r**) Resultant phase gradient of LCP and RCP component. The Fourier space spectrum (**s**) and real-space image (**u**) of a square object. (**s**) and (**u**) will be changed to (**t**) and (**v**), respectively, when a PB phase gradient metasurface is added at the Fourier plane. Blue- and red-shaded areas indicate the resultant edge information along the PB phase gradient direction.

The team also proposed an edge detection mechanism based on Pancharatnam–Berry phase (PB phase) metasurfaces [38]. They experimentally demonstrated broadband edge detection with high optical efficiency using the designed dielectric metasurface (Figure 16). These metasurfaces are fabricated by scanning a focused laser beam inside a glass substrate and can be easily integrated with traditional optical components. The proposed edge detection mechanism may find important applications in image processing, high-contrast microscopy, and real-time object detection in compact optical platforms such as mobile phones and smart cameras.

Compared to metallic metasurfaces, the optical efficiency for edge detection of the designed metasurface can be as high as 90%, owing to the relatively thick glass substrate with high transmission coefficients. It can also potentially realize any other PB phase gradient metasurface with high optical efficiency. Since the edge detection here is achieved through the interaction between different polarization components of light, this metasurface may also find applications in polarization-dependent high-contrast microscopy and compact optical processing systems.

**Figure 17:**
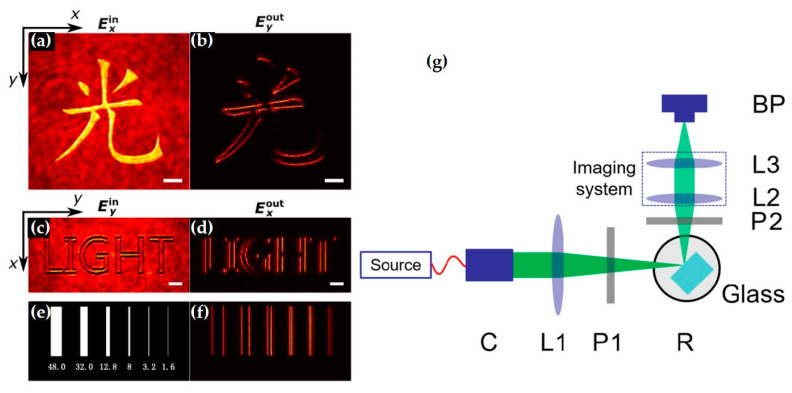
Spatial difference technique based on SHE [36]: (**a**) incident image consisting of the Chinese character for “light” with amplitude modulation on $E_{x}^{in}$; (**b**) reflected intensity image corresponding to (**a**) by measuring $E_{y}^{out}$; (**c**) incident image consisting of the LIGHT letters generated with phase modulation on $E_{y}^{in}$, where the inside and the outside of the letters have different phases but the same intensity; (**d**) reflected intensity image corresponding to (**c**) by measuring $E_{x}^{out}$. The white bars correspond to the length of 50 μm; (**e**) slot test patterns on the SLM with the different phases for the black and the white areas. The widths of the slots are listed below in micrometers; (**f**) measured reflected intensity image corresponding to (**e**); (**g**) Experimental setup for spatial differentiation under Gaussian beam illumination. Edge detection for different images stored in $E_{x}^{in}$ and $E_{y}^{out}$, respectively, with either amplitude or phase modulation.

Finally, they experimentally demonstrated the universality of spatial differentiation based on the SHE (Figure 17). The observation of spin-optical spatial differentiation depends on three key factors: oblique incidence, a coherent quasi-collimated beam, and a polarizer. Due to symmetry reasons, the SHE vanishes at normal incidence; therefore, the spatial differentiation signal weakens with a small value of $\left|r_{s}\;+\;r_{p}\right|$ as the incidence angle decreases. Furthermore, this spatial differentiation utilizes a simple and common structure to achieve vector field computation. Due to the universality of the SHE, the proposed method paves the way for ultrafast information processing in various optical systems and can even be applied to electron beam processing.

### 4.3. Optical Lateral Forces

Optical forces arise from the momentum exchange between light and matter; therefore, transverse momentum generates optical lateral forces. As a type of optical force, the underlying physical mechanisms are closely linked to many phenomena and effects: directional side scattering (DSS), spin–orbit coupling effects, surface plasmon polaritons, the imaginary Poynting vector momentum, and the transverse spin discussed in this paper.

A brief introduction to part of the theory of optical forces is given below. For a chiral Rayleigh particle, the optical force can be expressed as follows [18]:

(17) $$F=F_{grad}+F_{rad}+F_{curl}+F_{spin}+F_{flow}$$

(18) $$F_{\mathrm{grad}\;}=-\nabla U$$

(19) $$F_{\mathrm{rad}\;}=\frac{1}{c}\left(\sigma_{\mathrm{ext}\;}+\sigma_{\mathrm{recoil}\;}\right)P$$

(20) Fcurl =σpc∇×Se+σmc∇×Sm+μReαem(∇×P)

(21) Fspin=2ω2μReαem−k53πε2Imαeeαem*Se+2ω2μReαem−k5μ3πεImαmmαem*Sm

(22) Fflow =ck4μ212πImαeeαmm*ImE×H*

In the above equations, $\alpha_{ee}$ characterizes the electric dipole moment induced by an external electric field; $\alpha_{mm}$ characterizes the magnetic dipole moment induced by an external magnetic field; $\alpha_{em}$ quantifies the particle’s ability to exhibit magnetoelectric coupling. Here $\sigma_{p}$ and $\sigma_{m}$ represent equivalent cross-section parameters related to the electric and magnetic polarizabilities of the particle. σext=σp+σm=kIm(αee)/ε+kμIm(αmm) and σrecoil=−k4μ6πεRe(αeeαem∗)+αem2 are the extinction cross-section and recoil cross-section, respectively. $F_{grad}$ represents the optical gradient force, where *U* is given by

(23) U=−14Reαee|E|2−14Reαmm|B|2+12ReαemImB·E*

$F_{rad}$ describes the radiation pressure proportional to the time-averaged Poynting vector; $F_{curl}$ is the force generated by the curl of the spin and momentum, expressible as the curl of the SAM density; $F_{spin}$ originates from the coupling effect between particle chirality and the SAM density, thus existing only in chiral particles; $F_{flow}$ is related to the imaginary Poynting vector momentum.

From the above formulas, it can be seen that optical lateral forces can arise from various effects such as the Belinfante spin momentum, the imaginary Poynting vector momentum, and light–chirality interactions.

**Figure 18:**
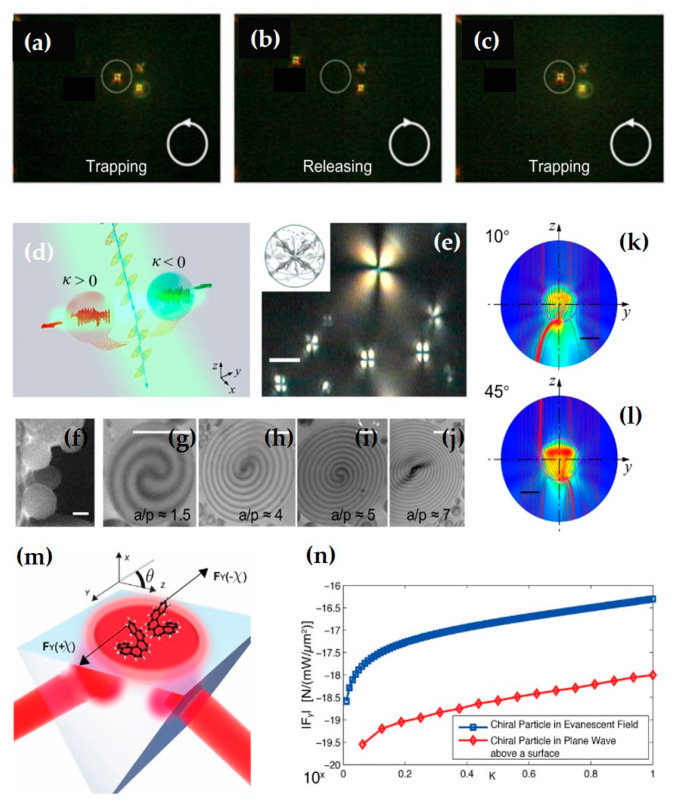
Generation and application of optical lateral forces: (**a**–**c**) optical gradient force on chiral particles. Experimental frames of the trapping, detrapping, and trapping of a cholesteric liquid crystal particle by switching the light polarization [188]; (**d**–**l**) experimental demonstration of the bidirectional enantioselective separation of a microsized cholesteric liquid crystal particle [189]. (**m**,**n**) Optical lateral force on the chiral particle in a linearly polarized evanescent wave [20].

The existence of optical lateral forces has been demonstrated in systems such as evanescent fields, transverse optical needle fields [182], the vicinity of an optical fiber [183,184], and Bessel beams [182]. Notably, as shown in Figure 18a–c, for chiral particles, their interaction with the optical field not only produces traditional optical lateral forces but also introduces a special optical lateral force component related to the t-SAM density. Due to the quantum SHE of light [78,185,186,187], the intrinsic t-SAM is locked to the propagation direction of surface plasmon polaritons, i.e., the spin-momentum locking effect [15,16,17], which indirectly gives the relationship between this optical lateral force component and the momentum density.

Research on the detection and application of optical lateral forces has mainly focused on particle manipulation. For example, Shi et al. from Tongji University placed cholesteric liquid crystal polymer particles of different chirality at the air–water interface [189]. As shown in Figure 18d–l, by impacting the particles with a linearly polarized beam, they observed bidirectional sorting of particles with different chirality. Furthermore, they found that the optical lateral force is closely related to particle size, chirality, incidence angle, and beam polarization state. Additionally, in coupled chiral particles, optical lateral forces can also exhibit binding, repulsion, and translation effects [190,191,192]. Figure 18m,n is a schematic diagram of the optical lateral force on a chiral particle in a linearly polarized evanescent wave. It should be noted that the optical lateral force arising from the t-SAM here can even be greater than that generated from the transverse energy flow (Poynting vector).

**Figure 19:**
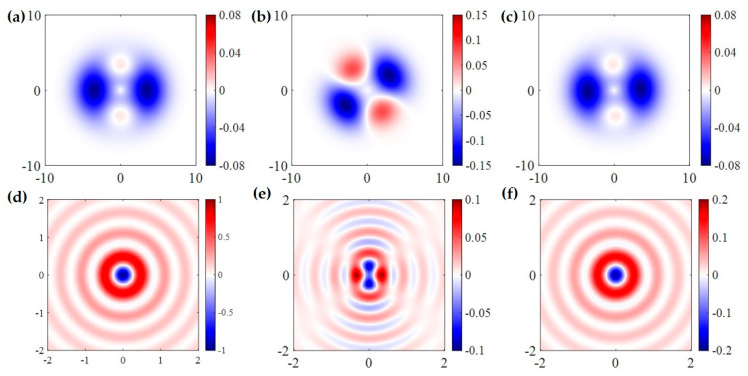
Optical backflow-induced lateral optical forces [193]: (**a**) azimuthal optical backflow generated by superposed OAM beams; (**b**) axial optical backflow generated by tightly focused cylindrical vector beams; (**c**,**d**) optical force distributions on dipolar nanoparticles showing local force reversal that does not coincide with the backflow region; (**e**,**f**) optimized material parameters leading to a force field consistent with the reversed energy flow.

In addition to chiral-induced lateral forces, optical backflow—a phenomenon in which the local energy flow is reversed—has recently been explored as an alternative mechanism for generating and controlling lateral optical forces. Xie et al. [193] demonstrated that such reversed energy flow can give rise to controllable lateral optical forces. As shown in Figure 19a,b, they used superpositions of OAM beams and tightly focused cylindrical vector beams to generate azimuthal and axial optical backflow, respectively. These backflow effects were further applied to the manipulation of dipolar nanoparticles to examine how the reversed energy flow influences the lateral optical forces. Interestingly, as shown in Figure 19c,d, the optical forces exhibited local reversal; however, their distribution did not coincide with the backflow region. By optimizing the particle’s material parameters, they subsequently achieved a force field consistent with the reversed energy flow, as illustrated in Figure 19e,f.

**Figure 20:**
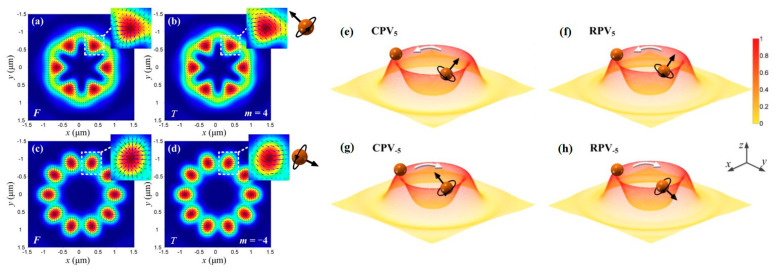
Optical lateral force and spin motion of different beams: (**a**–**d**) show the lateral force and spin torque distributions in the focal plane illuminated by vector vortex beams with topological charges (**a**,**b**) *m* = 4 and (**c**,**d**) *m* = −4 [194]; (**e**–**h**) show the spin and orbital motion of the particle illuminated by circularly polarized vortex beam inputs with topological charges of (**e**) *m* = 5 and (**g**) *m* = −5, and by radially polarized vortex beam inputs with (**f**) *m* = 5 and (**h**) *m* = −5 [195].

As shown in Figure 20a–d, this work [194] demonstrated that a tightly focused vector vortex beam with a petal-shaped intensity distribution and pure t-SAM can simultaneously trap multiple particles and cause them to rotate azimuthally. By changing the sign of the polarization topological charge, the direction of spin motion can be reversed. This work also pointed out that the spin torque decreases with increasing topological charge number and is also related to the numerical aperture of the objective lens, and the refractive index and absorption of the particle. As shown in Figure 20e–h, the research group also studied the focused fields of circularly polarized and radially polarized vortex beams [195], finding that the SAM and OAM carried by the beam can cause particles to perform non-axial spinning and orbital motion. The underlying optical torque responsible for these complex particle motions induced by light beam AM can be accurately calculated using classical methods based on Mie theory. This approach is not only applicable for analyzing the torque itself but has also been previously employed to measure minute optical absorption in particles. This suggests that, by utilizing custom-designed particles with known optical properties, the same methodology could be further developed into an effective means for detecting l-SAM and directly measuring its spatial distribution [91].

**Figure 21:**
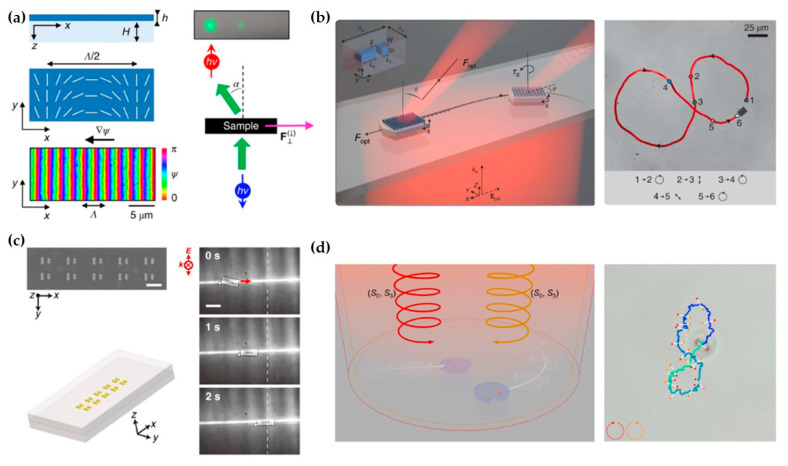
Microrobots based on OLFs: (**a**) one-dimensional microrobot which can move bilaterally with different helicity of light [196]; (**b**) one-dimensional linear microrobot by the OLF on a plasmonic nanoantenna, allowing the moving resolution to be beyond the diffraction limit [197]; (**c**) two-dimensional microrobot excited by both linear and CPL waves [198]; (**d**) three-dimensional light-driven microdrones using two overlapping light waves with two wavelengths [199].

Besides tightly focused beams, the optical lateral forces associated with structured beams such as Bessel beams, Airy beams, and vortex beams also offer many new possibilities for optical manipulation. The combination of these structured beams with subwavelength-scale metasurfaces has led to many novel applications. As shown in Figure 21a, by combining optical lateral forces with a one-dimensional structure designed via geometric phase, bidirectional movement of microrobots is achieved through the control of optical helicity [196]. Figure 21b shows a one-dimensional linear microrobot combined with a plasmonic nanoantenna, enabling displacement with a step size smaller than the diffraction limit [197]. Figure 21c shows a two-dimensional micromachine driven and controlled in its translation and rotation by both linearly polarized and circularly polarized beams [198]. Figure 21d shows a microdrone controlled by the superposition of two beams with different wavelengths [199].

### 4.4. Precision Sensing

**Figure 22:**
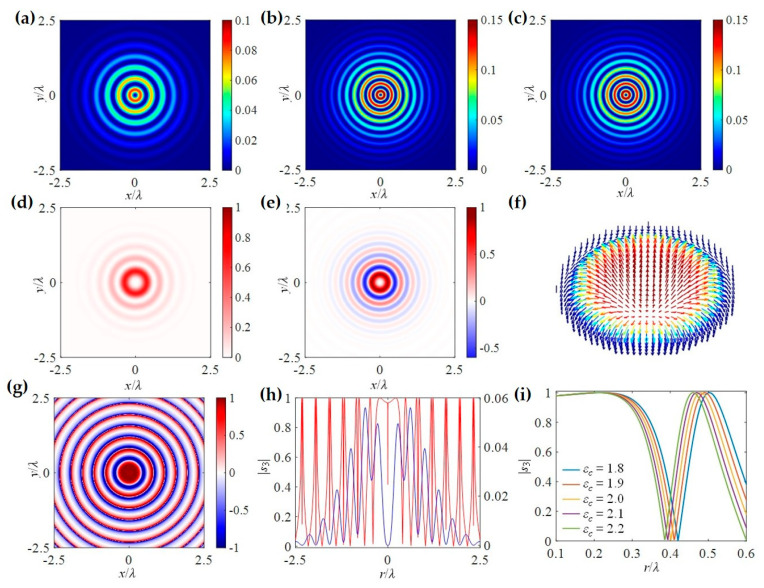
Simulated result of tunable optical spin skyrmions and spin fine structures at a metal/GRIN interface [200]: (**a**–**c**) electric field intensity distributions of the excited SPPs under different GRIN parameters: (**a**) $\varepsilon_{c}=2$, g= 2; (**b**)$\varepsilon_{c}=5$, g= 2; (**c**)$\varepsilon_{c}=5$, g= 5; (**d**–**i**) results obtained for GRIN parameters $\varepsilon_{c}=2$ and g= 2: radial and (**e**) axial (**z**) components of the SAM density; (**f**) normalized spin vector structure; (**g**) polarization ellipticity *s_3_*; (**h**) one-dimensional contour of $\left|s_{3}\right|$ and the corresponding horizontal field component ($E_{∥}$) along the radial direction; (**i**) gradient of the spin fine structure along the radial direction.

Optical SAM is also significant in the field of precision sensing. Therein, optical spin skyrmions have recently attracted significant attention for their potential in precision sensing owing to their topologically protected and spin fine structures. Theoretical simulations by Xie et al. investigated this potential by introducing graded refractive index materials (GRIN) into the near-field optical system, and analyzed surface plasmon polariton (SPP) propagation, enabling tunable optical skyrmions and spin fine structures at the metal/GRIN interface [200]. As shown in Figure 22a–c, tuning the parameters of the GRIN layer enables effective control of the excited SPP field, thereby enhancing its localization. Figure 22d–f illustrate the resulting SAM distributions and the skyrmion textures, revealing the topologically structured spin patterns at the interface. And, Figure 22g–i show the tunable spin fine structure with a minimal full-width of about 0.254 λ. Combined with a displacement platform, the theoretical precision corresponding to the minimal rotation angle can reach 2.54 × 10^−7^ λ (≈0.16 pm).

**Figure 23:**
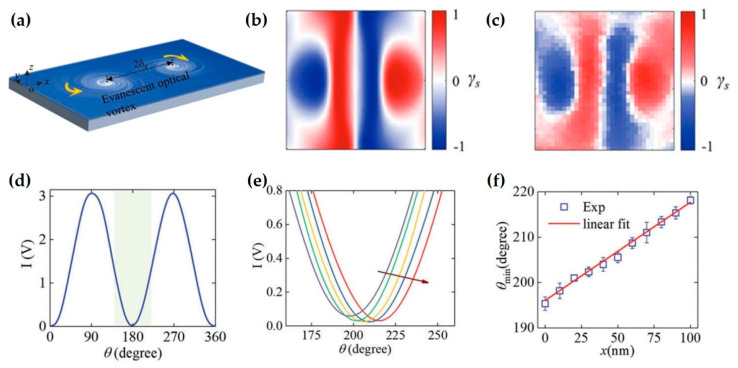
SAM applied to precision displacement sensing [29]: (**a**) illustration of the structured spin texture in a skyrmion-pair; (**b**,**c**) the spin distribution structure is obtained from $\gamma_{s}=(I_{RCP}-I_{LCP})/(I_{RCP}+I_{LCP})$. These two figures show the theoretical results and experimental measurements of the structure; (**d**,**f**) a typical response curves for displacement sensing; (**e**) illustrates the intensity curves and corresponding dip shifts measured via a rotating polarizer as the probe nanoparticle is displaced; (**f**) calibration curve of the dip angle versus displacement.

In experiment, Yang et al. proposed a method for manipulating photon spin using the spin-momentum equation [29] to study the spin texture in skyrmion pairs (Figure 23). They found that this texture exhibits sharply varying spin vectors at the nanoscale, enabling picometer-level displacement sensing. They theoretically designed a unique spin texture where the SAM varies linearly along the connection line between two skyrmions. By adjusting the spatial distance of the skyrmion pair, the linear range can reach 0.4 wavelengths, where the electric field intensity is maximized. Furthermore, by analyzing the Poynting momentum and spin structure of the skyrmion pair, they demonstrated that the transverse spin varies linearly only along the line connecting the centers of the two skyrmions and remains almost constant in the direction perpendicular to the connection line.

Therefore, within a 100-nanometer measurement range, the displacement sensing method based on this specially designed spin texture achieved picometer-level sensitivity. Since skyrmions are polarization-independent, future research might utilize transverse electric polarized evanescent waves with the same mechanism to construct other spin textures for novel displacement sensing technologies. In that case, the longitudinal electric field component is absent, and the detected transverse field strength is stronger, potentially further improving sensing stability. This spin-based manipulation method can provide new ideas for research related to spin photonics and could find applications in optical nanometrology, localization microscopy, and lithography mask alignment.

### 4.5. Magnetic Domain Detection

**Figure 24:**
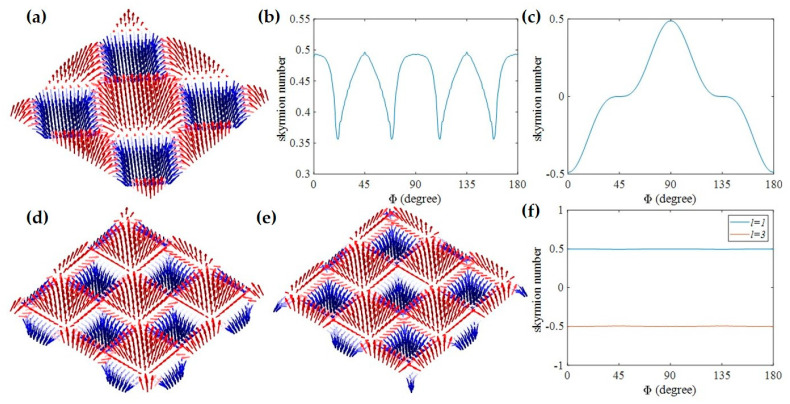
Simulated results of topological stability and transitions of photonic meron/skyrmion lattices at a metal/uniaxial interface [201]: (**a**) normalized real part of the out-of-plane electric-field component Re(Ez) at Φ = 0° with *l* = 0; (**b**,**c**) evolution of the skyrmion number within the central unit cell as a function of the optical-axis orientation Φ for *l* = 0 and *l* = 2, respectively; (**d**,**e**) present the normalized out-of-plane SAM distributions $S_{z}$ for *l* = 1 and 3 at Φ = 0°. (**f**) Variation in the skyrmion number within the unit cell at the center of the lattices via Φ.

Recent advances have demonstrated that topological spin structures, such as skyrmions and merons, are not merely subjects of fundamental interest but also possess remarkable sensitivity to the properties of their surrounding medium. In this context, Cao et al. theoretically investigated photonic meron lattices at a metal/uniaxial–crystal interface through numerical simulations [201]. By arranging surface plane waves with C_4_ symmetry, they found that when the total angular momentum (TAM = 0 or 2), the electric-field meron lattice is highly sensitive to the crystal’s optical-axis rotation. As shown in Figure 24a–c, rotating the crystal converts the SPP mode from a pure TM to a hybrid TE–TM state, inducing a topological phase transition and enabling controllable switching between nontrivial and trivial states. In contrast, for TAM = 1 and 3 (Figure 24d–f), the spin-meron lattices remain topologically invariant, as the decomposition of TAM into orbital and spin components in an anisotropic medium is analogous to that in an isotropic one. This phenomenon indicates that, under specific symmetry and SAM configurations, photonic meron lattices possess both reconfigurability and topological robustness, providing a theoretical foundation for constructing tunable photonic spin topologies.

**Figure 25:**
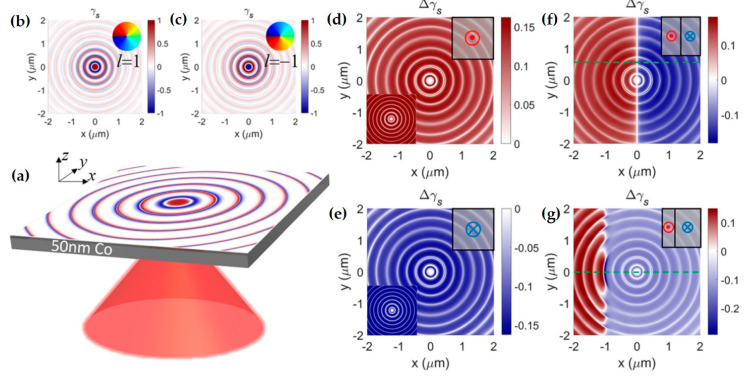
Optical spin–orbit coupling effect applied to magnetic domain detection [30]: (**a**) schematic diagram for the generation of a photonic skyrmion in a ferromagnetic material on the example of the SPP excited on a surface of a thin (50 nm) Co film by a tightly focused (NA = 1.49) radially polarized beam; (**b**,**c**) spin states of (**b**) positive and (**c**) negative skyrmions generated with l=±1 RP beam, respectively, with the Co magnetization in a positive *z* direction; (**d**) spatial distribution of $\Delta \gamma_{s}$ for the skyrmions in (**b**,**c**). (**e**) The same as (**f**) for the magnetization oriented in negative *z* direction. (**f**,**g**) Spatial distributions of $\Delta \gamma_{s}$ for the magnetic structure consisting of two domains with opposite magnetization orientation.

These findings offer new perspectives for the optical detection of magnetic domain structures. Since the magnetization state can modulate the photonic spin distribution, thereby influencing the morphology and evolution of topological spin structures, the sensitivity of photonic meron lattices to interface medium parameters can be leveraged to develop magnetic domain imaging techniques based on spin topology variations. This enables non-destructive detection of magnetic domain states at deep-subwavelength scales. Lei et al. studied the interaction between optical spin–orbit coupling and photonic skyrmions with magnetic domains under magnetization conditions [30], demonstrating that magnetization can alter the photonic spin distribution, consequently generating twisted Néel-type skyrmions (Figure 25). This effect can reflect the in-plane or out-of-plane magnetic domain structure. Conversely, magnetization patterns can also be utilized to create complex optical spin distributions. The interaction between skyrmions and magneto-optical effects, as demonstrated by them, opens up new possibilities for studying and manipulating magnetic domains using optical spin–orbit coupling.

Nanoscale imaging of magnetic domain structures is crucial for magnetic storage technology and spintronics. Conventional optical detection methods, such as the magneto-optical Kerr effect, are limited by the diffraction limit. Wu et al. proposed a novel approach by leveraging a topological meron spin lattice formed on a hyperbolic metamaterial (HMM) surface as a deep-subwavelength probe. By measuring the differential spin distribution signals under excitation with different circularly polarized lights, they achieved the detection of arbitrary polar magnetization domains. This work transforms photonic topological quasiparticles from subjects of study into powerful probing tools, offering a new solution for the characterization of next-generation high-density magnetic memory devices.

**Figure 26:**
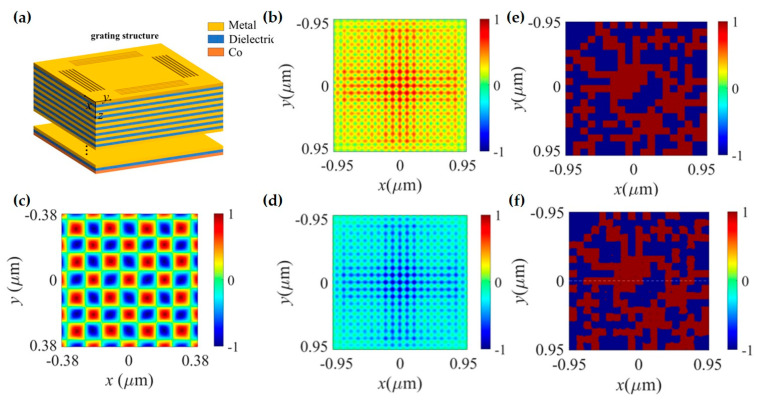
Optical spin meron lattice applied to deep-subwavelength magnetic domain detection. (**a**) Schematic diagram of the magnetic domain sensor based on a HMM; (**b**) out-of-plane spin component ($S_{z}$) distribution of the optical meron spin lattice on the HMM surface, obtained by finite-difference time-domain (FDTD) simulations. The lattice unit cell size is 100 nm, exhibiting alternating “up” and “down” spin states; (**c**,**d**) the spin distribution signal ($\Delta S_{z}=\hat{T}(S_{z}^{LCP})+S_{z}^{RCP}$) obtained by superposing the spin distributions excited by left-handed and right-handed circularly polarized light when the magnetization direction is +z and −z. Its positive or negative sign directly corresponds to the magnetization direction of the magnetic domains; (**e**,**f**) the preset random magnetic domain matrix, the experimentally detected binarized result, and the detection error distribution. The results demonstrate magnetic domain reconstruction with a resolution of 100nm and 100% accuracy under these parameters.

Besides the aforementioned examples of SAM applications, our research group is actively exploring the application of photonic spin structures in other fields, including the detection of fractional OAM and the detection of the refractive index of biological media (Figure 26). These two research efforts demonstrate the applicability and potential value of photonic spin structures in practical applications, marking a further expansion of our exploration into the application areas of photonic spin structures.

## 5. Summary and Outlook

As a degree of freedom in optics following intensity, phase, and polarization, the near-field information carried by SAM has broad application prospects in fields such as communication, imaging, and precision detection. In this article, we have introduced the concept, definition, classification, and physical origin of SAM, reviewed the detection methods developed in recent years, and its applications in weak effect detection, optical differentiation, optical lateral forces, precision sensing, and magnetic domain detection. On one hand, as a novel optical degree of freedom, SAM can provide new solutions for large-scale light-field manipulation and optical communication applications. On the other hand, SAM is a fundamental dynamical physical quantity of basic particles like photons and atoms, offering new perspectives for small-scale light–matter interactions, optical imaging, and optical detection. Consequently, it can further play a significant role in exploring new mechanisms and phenomena in light–matter interactions and expanding the applications of spin photonics.

## Figures and Tables

**Table 1:** Dynamical and topological properties of general electromagnetic waves, linearly polarized surface electromagnetic waves, deep-water gravity waves, and acoustic wave fields.

	Generic EM Wave	Linear Polarized Surface EM Wave	Gravity Water Wave	Acoustic Wave
Field	Electric field E; Magnetic field H;	Electric or magnetic Hertz potential Ψ;	In-plane velocity V; Normal velocity W;	Velocity v; Pressure p;
PM	Π=12c2ReE∗×H	Π=εk2kp22ωImΨ∗∇Ψ	ΠG=ρGkGωGImW∗V	ΠA=12cA2Rep∗v
SAM	S=14ωImεE∗×E+μH∗×H	S=εkp24ωIm∇Ψ∗×∇Ψ	SG=ρG2ωGImV∗×V	SA=ρA2ωAImv∗×v
Helicity	Spin-1 photon σ=±1	Spin-1 photon σ=±1	Spin-0 phonon $\sigma_{G}=0$	Spin-0 phonon $\sigma_{A}=0$
iSML	$\begin{array}{c} S_{t}=\frac{1}{2k^{2}}\nabla \times \Pi \\ S_{l}=\sum_{i}\hbar \sigma_{i}{\hat{k}}_{i}+\sum_{i\neq j}\hbar \sigma_{ij}{\hat{k}}_{ij} \end{array}$	$\begin{array}{c} S_{t}=\frac{1}{2k^{2}}\nabla \times \Pi \\ S_{l}=0 \end{array}$	$S_{G}=\frac{1}{2k_{G}^{2}}\nabla_{2}\times \Pi_{G}$	$S_{A}=\frac{1}{k_{A}^{2}}\nabla \times \Pi_{A}$

## Data Availability

No new data were created or analyzed in this study.
